# Identification and validation of the association of Janus kinase 2 mutations with the response to immune checkpoint inhibitor therapy

**DOI:** 10.1007/s00011-023-01833-w

**Published:** 2024-01-10

**Authors:** Peipei Chen, Junyu Long, Jiayang Zhang, Fucun Xie, Wei Wu, Zhuang Tian, Shuyang Zhang, Kang Yu

**Affiliations:** 1 Department of Clinical Nutrition & Health Medicine, Peking Union Medical College Hospital, Chinese Academy of Medical Sciences and Peking Union Medical College, Beijing, China; 2 Department of Liver Surgery, State Key Laboratory of Complex Severe and Rare Diseases, Peking Union Medical College Hospital, Chinese Academy of Medical Sciences & Peking Union Medical College, Beijing, China; 3 Key Laboratory of Carcinogenesis and Translational Research (Ministry of Education/Beijing), Department of Breast Oncology, Peking University Cancer Hospital & Institute, Beijing, China; 4 Department of Cardiology, Union Medical College Hospital, Chinese Academy of Medical Sciences and Peking Union Medical College, Beijing, 100730 China

**Keywords:** Janus kinase 2, Biomarker, Immune response, Immune checkpoint inhibitor, Immunotherapy, Prognosis, Tumor microenvironment

## Abstract

**Background:**

Janus kinase 2 (JAK2) mutation plays an important role in T cell immunity. However, the effect of JAK2 mutation on immunotherapy is largely uncharacterized.

**Methods:**

In this study, we analyzed the effect of JAK2 mutation on the efficacy and outcomes of immune checkpoint inhibitor (ICI) therapy in the discovery cohort (*n* = 662) and the verification cohort (*n* = 1423). Furthermore, we explored the association of JAK2 mutation with the tumor immune microenvironment in a multiomics cohort.

**Results:**

In the discovery cohort (*n* = 662), JAK2 mutant-type patients had a better objective response rate (58.8% vs. 26.7%, *P* = 0.010), durable clinical benefit (64.7% vs. 38.9%, *P* = 0.043), progression-free survival (hazard ratio [HR] = 0.431, *P* = 0.015), and overall survival (HR = 0.378, *P* = 0.025), relative to JAK2 wild-type patients. Moreover, we further verified the prognostic significance of JAK2 mutation in an independent ICI treatment cohort with a larger sample size (*n* = 1423). In addition, we discovered that the JAK2 mutation was remarkably related to increased immunogenicity, such as a higher TMB, higher expression of costimulatory molecules and stimulation of antigen processing mechanisms. In addition, JAK2 mutation was positively correlated with activated anticancer immunity, such as infiltration of various immune cells and higher expression of chemokines.

**Conclusion:**

Our study demonstrates that JAK2 mutation is a novel marker that can be used to effectively predict prognosis and response to ICI therapy.

**Supplementary Information:**

The online version contains supplementary material available at 10.1007/s00011-023-01833-w.

## Highlights

JAK2-mutated patients had a better response to ICI therapy.

JAK2 mutation was associated with higher levels of immune cell infiltration and immune checkpoints.

JAK2 mutation may be served as a potential predictive biomarker for efficacy of ICI therapy.

**Supplementary Information:**

The online version contains supplementary material available at 10.1007/s00011-023-01833-w.

## Introduction

A substantial number of biomarkers are associated with the tumor immune checkpoint inhibitor (ICI) response. Among these markers, the tumor mutation burden (TMB), programmed death ligand 1 (PD-L1), and deficient mismatch repair (dMMR)/high microsatellite instability (MSI-H) have been validated in phase III clinical trials [1–3]. However, many challenges remain for their practical clinical application due to intratumoral and intertumoral heterogeneity. For example, PD-L1 levels have different positive reference thresholds in different tumor types. Nontissue-related biomarkers, such as a high TMB (TMB-H) and dMMR/MSI-H, lack universal standards. Given the complexity of the immune system, a single biomarker may not be sufficient to accurately predict a therapeutic benefit, and biomarkers to effectively predict the outcome of ICI treatment are lacking [4]. Therefore, considering the high cost and potential immune-related adverse events of ICI treatment, the identification of additional predictive markers with high specificity and sensitivity to predict which patients will benefit from ICIs is imperative.

The Janus kinase (JAK) protein family shares functional and functional homologies, and its members consist of TYK2, JAK3, JAK2, and JAK1 [5]. An abnormal JAK2 expression pattern has been observed in various types of tumors [6]. The JAK2-signal transducer and activator of transcription 3 (STAT3) signaling pathway is a universal intracellular pathway that plays an important role in many biological processes, such as immune regulation, cell apoptosis, differentiation, and proliferation [5]. JAK2 activation induces constitutive phosphorylation of STAT3, potentially driving neoplasm growth and metastasis [7]. In addition, inhibition of JAK2 suppresses the proliferation and migration of gastric cancer [8], breast cancer [9, 10], and pancreatic cancer [11] cells. In animal models, simultaneous inhibition of PI3K/mTOR and JAK2 reduces tumor growth [9]. Both PD-L1 and JAK2 are located on chromosome 9p.24 [12]. In the tumor microenvironment, tumor cells suppress T-cell function by increasing PD-L1 expression through JAK–STAT signaling [13]. Prestipino et al. found that PD-L1 surface expression was overexpressed on monocytes and megakaryocytes from JAK2 mutant mice compared with JAK2 wild-type littermate control mice [12]. Furthermore, PD-L1-expressing JAK2 mutant cells affect the metabolism and cell cycle progression of T cells [12]. However, the role of JAK2 in the human tumor immune microenvironment has not been comprehensively studied, and the relationship between JAK2 mutation and the immunotherapy response is unclear.

In this study, we explored JAK2 mutations in publicly available clinical and genomic datasets to evaluate their correlations with the immune microenvironment and response to ICI therapy. In patients with various types of malignances, JAK2 mutation indicated an improved progression-free survival (PFS), overall survival (OS), objective response rate (ORR), and durable clinical benefit (DCB) of ICI treatment.

## Materials and methods

### Clinical cohort of patients

We collected a discovery cohort of patients with mutation data and outcome data who received ICI treatment from seven published studies to assess the prognostic significance of JAK2 mutations (Figure S1A) [14–20]. The mutation data of six published studies [14–19] obtained after processing were acquired from cBioPortal (https://www.cbioportal.org) and the mutation data of the study by Liu et al. were acquired from the attachment to the previous article [20]. All nonsynonymous somatic mutations in JAK2, including translational start site, splice site, nonstop, nonsense, frame-shift, and missense mutations, were considered [21]. Cancers with and without nonsynonymous somatic mutations of JAK2 were considered JAK2-mutated (JAK2-mutated) and JAK2-wild-type (JAK2-wild) cancers, respectively. Two cohorts were sequenced using the Memorial Sloan Kettering-Integrated Mutation Profiling of Actionable Cancer Targets (MSK-IMPACT) panel [14, 15], and the remaining five cohorts were sequenced using whole-exome sequencing (WES) [16–20]. Notably, three cohorts [16–18] were previously curated and filtered based on standard quality control measures to ensure adequate power to detect tumor-specific mutations by Miao et al. [19]. Through the result [19] by Miao et al.'s screening of other researchers' cohorts [16–18] and their own cohort [19], we further discarded tumor types with insufficient sample sizes (< 10). Ultimately, the discovery cohort included 662 patients with five tumor types: melanoma (*n* = 287), non-small cell lung cancer (NSCLC, *n = *296), head and neck squamous cell carcinoma (HNSCC, *n* = 12), esophagogastric carcinoma (*n* = 40), and bladder carcinoma (*n* = 27).

In addition, we verified the prognostic significance of JAK2 mutations in the verification cohort, an independent ICI-treated cohort from the study by Samstein et al. (*n* = 1423) comprising patients with NSCLC (*n* = 157), melanoma (*n* = 320), renal cell cancer (*n* = 151), colorectal cancer (*n* = 109), esophagogastric carcinoma (*n* = 88), glioma (*n* = 117), bladder cancer (*n* = 215), HNSCC (*n* = 138), carcinoma of unknown origin (*n* = 84), and breast cancer (*n* = 44) with survival data but lacking response data (Fig. S1B) [22]. The mutation data obtained from patients after ICI treatment by Samstein et al. were acquired from cBioPortal (https://www.cbioportal.org).

A non-ICI cohort (*n *= 3791) from the study by Zehir et al. was analyzed to confirm that the association of JAK2 mutations with a better prognosis was due to ICI treatment, rather than a general prognostic benefit (Fig. S1C) [23]. The mutation data obtained from the study by Zehir et al. were acquired from cBioPortal (https://www.cbioportal.org).

Moreover, the mutational and expression data from patients with 33 types of tumors in TCGA cohort, which were acquired from the PanCancer Atlas Consortium (https://gdc.cancer.gov/about-data/publications/pancanatlas), were utilized to explore the different immune response features of JAK2-wild and JAK2-mutated cancers.

### Determination of the TMB

For specimens sequenced using the MSK-IMPACT panel, the sum of nonsynonymous variants was normalized to the exonic coverage of the MSK-IMPACT panel (0.98, 1.06, and 1.22 Mb in the 341-, 410-, and 468-gene panels, respectively). For specimens sequenced using WES, the TMB was calculated as the sum of nonsynonymous variants divided by the exome size (38 Mb).

### Estimation of clinical results

As described in a previous study [15], we utilized Response Evaluation Criteria in Solid Tumors (RECIST) 1.1 to evaluate the ORR. Patients were characterized as having no durable benefit (NDB, stable disease [SD] lasting ≤6 months, or disease progression [PD]) or DCB (complete response [CR]/partial response [PR] or SD lasting > 6 months) [15]. In the non-ICI cohort, OS was determined from the procedure date when the cancer sample was harvested to the date of death or the last follow-up [23]. In the ICI cohort, OS was determined from the start date of ICI treatment to the date of death or last follow-up.

### CIBERSORT analysis of immune cell infiltration

The CIBERSORT algorithm for determining the proportions of infiltrating immune cells was obtained from the pancancer immunity landscape project (PILP) by Thorsson et al. [24]. CIBERSORT is a gene expression-related deconvolutional algorithm that employs the support vector regression method to infer cell type percentages [25]. The percentages of 22 types of infiltrating immune cells were computed with the CIBERSORT approach.

### Analyses of tumor-infiltrating lymphocytes (TILs)

The genome data-derived proportions of TILs and the H proportions of TILs derived from H&E staining of samples from TCGA pancancer cohort were derived from the studies by Thorsson et al. and Saltz et al., respectively [24, 26]. The genome data-derived proportion of TILs was attained by multiplying the proportion of leukocytes estimated from the DNA methylation data by the proportion of lymphocytes estimated from the expression data [24].

### Evaluation of immune signatures

In this study, 29 typical immune hallmarks were acquired from previously published research [27]. The enrichment levels of the 29 immune hallmarks in every specimen were quantified using single-sample gene set enrichment analysis (ssGSEA) with the “GSVA” R package (version 1.46.0) in R software (version 4.2.1) [28].

### Analysis of the cytolytic (CYT) score

The CYT score was computed as the geometric average of the expression of granzyme A (GZMA) and perforin 1 (PRF1) [29].

### Comprehensive evaluation of immunophenotyping

Immunogenomic indicators, including non-silent mutation rate, silent mutation rate, single-nucleotide variant (SNV) neoantigens, indel neoantigens, aneuploidy score, fraction altered, T-cell receptor (TCR) richness, and B-cell receptor (BCR) richness, were obtained from the study by Thorsson [24]. In addition, we collected interferon-γ (IFN-γ) and T-cell-inflammatory gene expression profile (GEP) from the study by Ayers [7] and cancer immune cycle-related genes from the study by Chen [30]. The enrichment levels of the IFN-γ, GEP, and cancer immune cycle in every specimen were quantified using ssGSEA with the “GSVA” R package (version 1.46.0) in R software (version 4.2.1).

### JAK2 mutations mapped to biological functions

Investigating JAK2 mutation-related functional pathways and molecular mechanisms facilitates a better understanding of the underlying biological dissimilarities between JAK2-wild and JAK2-mutated patients. First, the difference analysis was conducted between JAK2-wild and JAK2-mutated patients using the “limma” package (version 3.54.0). Then, the top 100 highly expressed genes in the JAK2-wild and JAK2-mutated patients were analyzed using the “clusterProfiler” R package (version 4.6.0) for enriched functions and pathways.

### Statistics

Fisher’s exact test was utilized to evaluate the relationships of JAK2 with outcomes (ORR and DCB). The statistical analysis of the difference between 2 groups was performed using the Wilcoxon method. The log-rank test and Cox proportional risk regression analyses were employed to study the differences in PFS and OS between JAK2-mutated and JAK2-wild patients. All statistical analyses were performed with R software version 4.2.1, and the *P* values were calculated using two-tailed tests. *P* < 0.05 indicated statistical significance.

## Results

ICIs have produced a significant clinical benefit in patients with cancers, but only a subset of patients respond to current immunotherapeutic strategies. The identification of patients who may benefit from ICI therapy is urgently needed to improve the response rate to ICI therapy. In this study, we sought to define the relationships between JAK2 mutation and the response to ICI therapy and immunogenic profile of cancers. We found that JAK2 mutation independently and stably predicted the prognosis of patients with cancer who were treated with ICI therapy, and it is considered a promising biomarker to identify patients suitable for ICI therapy. JAK2-mutated patients showed an immunologically active phenotype, including higher infiltration of various immune cells and enhanced immunogenicity.

### Association of JAK family mutations with the response to ICI treatment

**Fig. 1:**
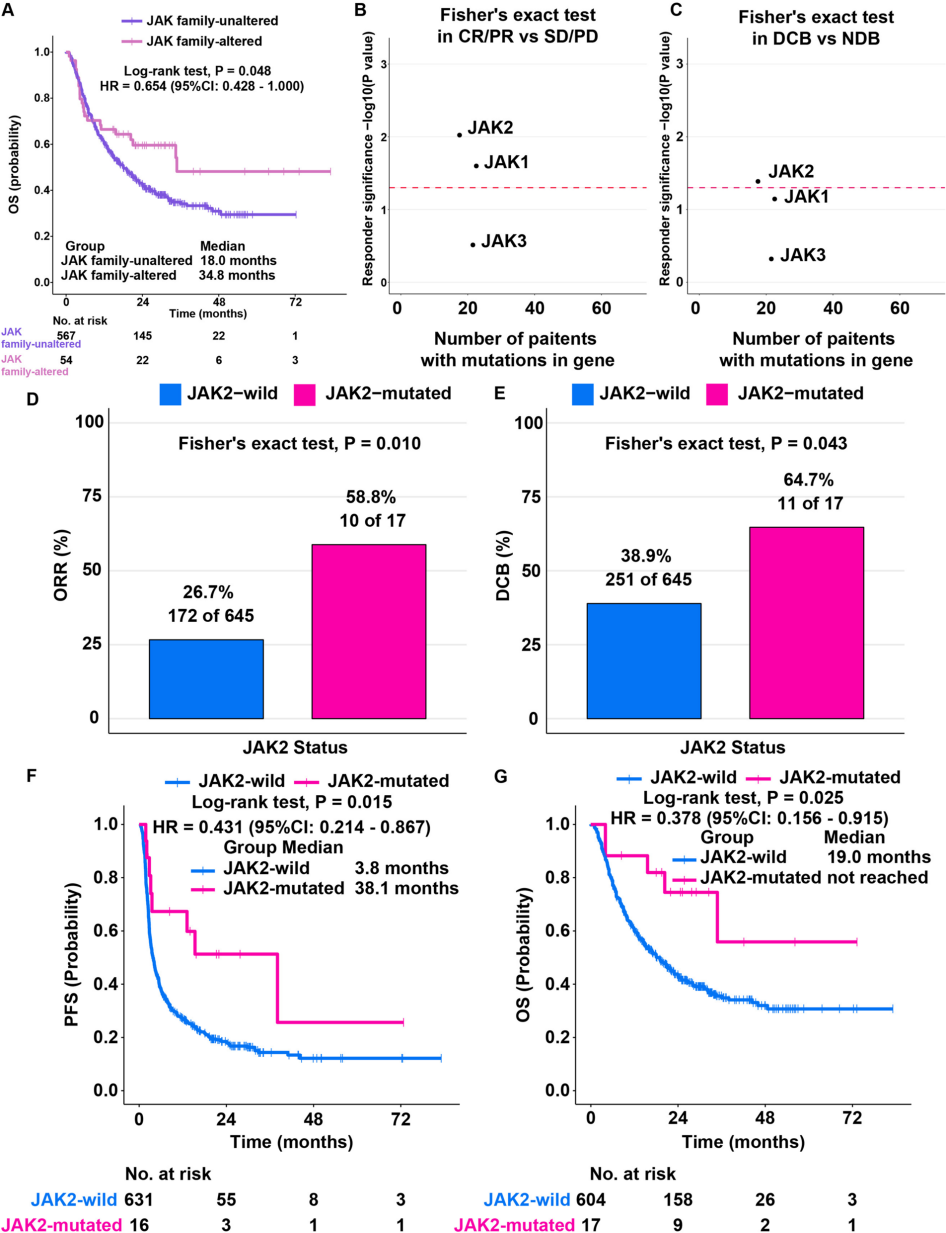
Overall survival of JAK2-wild and JAK2-mutated patients treated with ICIs.** A** Kaplan–Meier curves of OS were compared among JAK family-altered and JAK family-unaltered patients. **B** The response rates were compared among JAK1, JAK2, and JAK3-mutated patients. The dashed red line indicates *P* = 0.05. **C** The clinical benefits were compared among JAK1-, JAK2-, and JAK3-mutated patients. The dashed red line indicates *P* = 0.05. **D** The response rates were compared between JAK2-wild and JAK2-mutated patients. **E** The clinical benefits were compared between JAK2-wild and JAK2-mutated patients. **F** Kaplan–Meier curves of PFS were compared between JAK2-wild and JAK2-mutated patients. **G** Kaplan–Meier curves of OS were compared between JAK2-wild and JAK2-mutated patients

We integrated the outcome and mutation data from seven ICI-treated cohorts into a discovery cohort, which included 662 patients with five types of tumors: esophagogastric cancer (*n* = 40), NSCLC (*n* = 296), melanoma (*n* = 287), bladder carcinoma (*n* = 27), and HNSCC (*n* = 12) (Fig. S1A). Four mammalian JAKs have been identified, JAK1, JAK2, JAK3, and TYK2. However, TYK2 does not exist in the MSK-IMPACT panel, so we defined that as long as one of JAK1, JAK2, and JAK3 gene was mutated, it was defined as a mutation in JAK family. We found that patients with JAK family mutation (median OS: 34.8 months) experienced a longer OS than patients without JAK family mutation (median OS: 18.0 months) within the discovery cohort (hazard ratio [HR] = 0.654, *P* = 0.048, Fig. 1A). Moreover, JAK2 mutation was significantly associated with better ORR (*P* < 0.05, Fig. 1B) and DCB (*P* < 0.05, Fig. 1C), suggesting that JAK2 mutation might be a marker for predicting ICI treatment outcomes.

### JAK2 mutation was associated with superior clinical ICI treatment outcomes

According to RECIST 1.1, the outcome data for the 662 patients within the discovery cohort were assessed. The ORR of JAK2-mutated patients was over two times greater than that of JAK2-wild patients (58.8% versus 26.7%, Fig. 1D). In addition, 64.7% of patients with JAK2 mutations acquired DCBs, compared with merely 38.9% of JAK2-wild patients (*P* = 0.043, Fig. 1E). As anticipated, in contrast to JAK2-wild patients (*n* = 61), JAK2-mutated patients experienced a longer PFS (*n *= 631, HR = 0.431, *P* = 0.015, Fig. 1F). The median PFS was 38.1 months for JAK2-mutated patients compared with 3.8 months for JAK2-wild patients. The OS was also higher in JAK2-mutated patients (the median OS was not reached) than in JAK2-wild patients (median OS: 19.0 months) (HR = 0.378, *P* = 0.025, Fig. 1G). Hence, JAK2 mutation might be a predictive factor, and JAK2-mutated patients may derive greater benefits from ICI therapy.

### The JAK2 mutation status was an independent predictive factor of immunotherapy outcomes

Univariable and multivariable Cox regression analyses were performed to assess the independent prognostic significance of JAK2. Univariable Cox regression analyses of PFS for age, gender, cancer type, drug type, and JAK2 mutation status revealed that the JAK2 mutation status and tumor type were prognostic factors. Therefore, the JAK2 mutation status and tumor type were subjected to multivariable Cox regression analyses of PFS. Multivariable Cox regression analyses revealed that the JAK2 mutation status was an independent prognostic factor related to PFS. Similar outcomes were observed in the univariable and multivariable Cox regression analyses of OS. After adjustment for tumor type, the JAK2 mutation status remained an independent prognostic factor related to OS.

### Verification of the prognostic significance of the JAK2 mutation status

An independent ICI-treated cohort (*n* = 1423) was studied to confirm the association between JAK2 mutation and OS and to verify the significance of the JAK2 mutation status in predicting the OS benefit (Fig. S2A). In this verification cohort, JAK2-mutated patients (the median OS was not reached) experienced remarkably longer OS than JAK2-wild patients (median OS: 19.0 months) (HR = 0.443, *P* = 0.025, Fig. S2A). In addition, we verified that the OS benefits of ICI treatment in JAK2-mutated patients were not a result of general effects of the mutation on prognosis by evaluating the difference in OS between JAK2-mutated and JAK2-wild patients within a non-ICI-treated cohort (*n* = 3791) (Fig. S2B). No difference in OS was observed between JAK2-mutated patients (the median OS was not reached) and JAK2-wild patients (median OS: 25.4 months) within the non-ICI-treated cohort (HR = 1.086, *P* = 0.728, Fig. S2B).

### Potential intrinsic immune response mechanisms of JAK2-mutated and JAK2-wild cancers

**Fig. 2:**
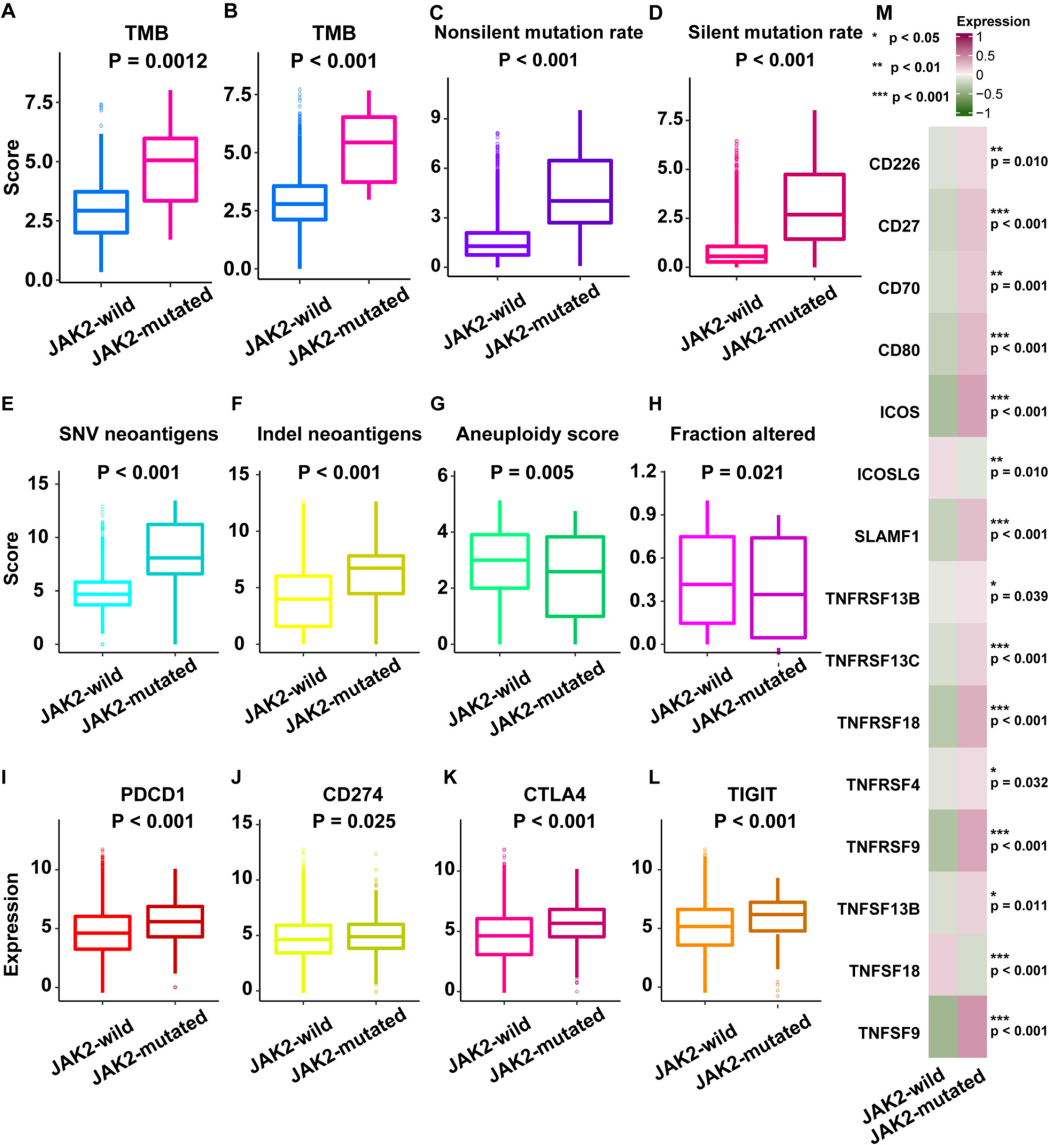
The landscape of immunogenicity of JAK2-wild and JAK2-mutated patients. **A** The TMB was compared between JAK2-wild and JAK2-mutated patients in the discovery cohort. **B** The TMB was compared between JAK2-wild and JAK2-mutated patients in the validation cohort. **C** The non-silent mutational rate was compared between JAK2-wild and JAK2-mutated patients in TCGA cohort. **D** The silent mutational rate was compared between JAK2-wild and JAK2-mutated patients in TCGA cohort. **E** SNV neoantigens were compared between JAK2-wild and JAK2-mutated patients in TCGA cohort. **F** Indel neoantigens were compared between JAK2-wild and JAK2-mutated patients in TCGA cohort. **G** The aneuploidy score was compared between JAK2-wild and JAK2-mutated patients in TCGA cohort. **H** The CNV burden was compared between JAK2-wild and JAK2-mutated patients in TCGA cohort. **I** PDCD1 expression was compared between JAK2-wild and JAK2-mutated patients in TCGA cohort. **J** CD274 expression was compared between JAK2-wild and JAK2-mutated patients in TCGA cohort. **K** CTLA4 expression was compared between JAK2-wild and JAK2-mutated patients in TCGA cohort. **L** TIGIT expression was compared between JAK2-wild and JAK2-mutated patients in TCGA cohort. **M** The expression of costimulatory molecules was compared between JAK2-wild and JAK2-mutated patients in TCGA cohort

The intrinsic immune responses of JAK2-mutated and JAK2-wild cancers were explored to elucidate the mechanisms underlying the prognostic significance of JAK2 mutations in the response to ICI treatment. In contrast to JAK2-wild cancers, the TMB in JAK2-mutated cancers was remarkably greater in the discovery cohort (*P* = 0.0012, Fig. 2A) and within the verification cohort (*P* < 0.001, Fig. 2B). Moreover, we utilized TCGA cohort multiomics data to investigate the differences in the immune landscape between JAK2-mutated and JAK2-wild cancers. In contrast to those in JAK2-wild cancers, the mutation rate (both the non-silent and silent mutational rates) (*P* < 0.001, Fig. 2C and 2D) and neoantigen load (including SNV-derived neoantigens and indel-derived neoantigens) (*P* < 0.001, Fig. 2E, F) were remarkably greater in JAK2-mutated cancers, revealing that JAK2 mutation was associated with increased cancer immunogenicity. Compared with JAK2-wild tumors, JAK2-mutated tumors exhibited a higher rate of aneuploidy (*P* = 0.005) (Fig. 2G) and a higher copy-number variation (CNV) burden (*P* = 0.021) (Fig. 2H). These results are consistent with the previous discovery that tumor aneuploidy is related to a reduced response to immunotherapy and to markers of immune evasion [31].

**Fig. 3:**
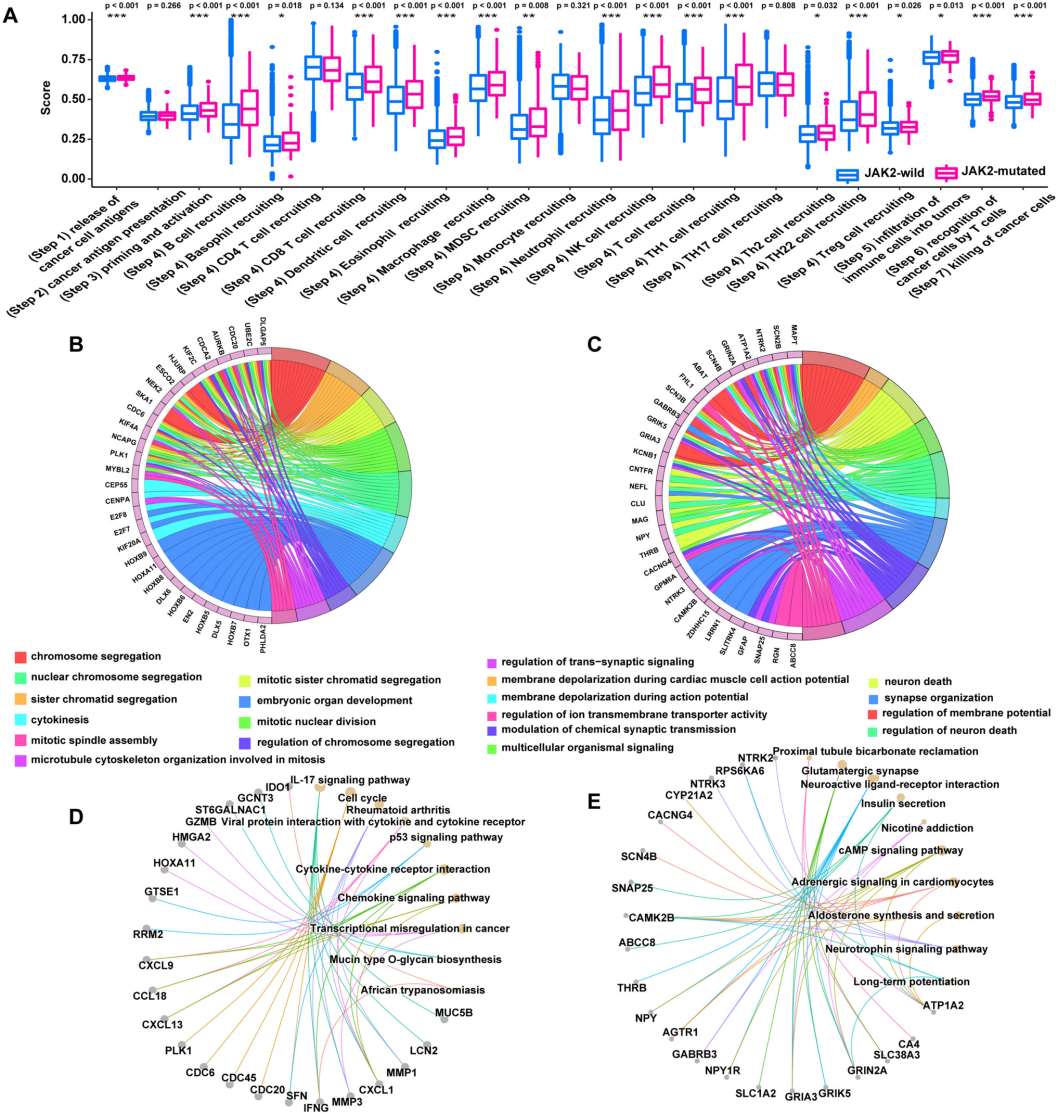
The potential mechanisms in JAK2-wild and JAK2-mutated patients. **A** The cancer immune cycles were compared between JAK2-wild and JAK2-mutated patients in TCGA cohort. **B** Functional analysis for top 100 high expression genes of JAK2-mutated patients in TCGA cohort. **C** Functional analysis for top 100 high expression genes of JAK2-wild patients in TCGA cohort. **D** Pathway analysis for top 100 high expression genes of JAK2-mutated patients in TCGA cohort. **E** Pathway analysis for top 100 high expression genes of JAK2-wild patients in TCGA cohort

We discovered that JAK2-mutated cancers displayed upregulated expression of immune checkpoint molecules (such as PDCD1, CD274, CTLA4, and TIGIT) compared with JAK2-wild tumors (Fig. 2I–L). Another vital factor potentially affecting the intrinsic immune response is the expression of costimulatory molecules. The expression of some costimulatory molecules was higher in JAK2-mutated cancers than in JAK2-wild cancers (Fig. 2M). The cancer immune cycle-related genes were extracted from the study by Xu [30, 32]. We used the GSVA method to evaluate the cancer immune cycle and found that JAK2-mutated patients had higher expression of many cancer immune cycles, including those related to (step 1) the release of cancer cell antigens, (step 3) priming and activation, (step 4) trafficking of immune cells to tumors, (step 5) infiltration of immune cells into tumors, (step 6) recognition of cancer cells by T cells, and (step 7) killing of cancer cells, than JAK2-wild patients (Fig. 3A), indicating that cancer immune cycles may be more active in patients with JAK2 mutations. Based on these results, JAK2-mutated cancers feature enhanced cancer immunogenicity and a relatively hot immune microenvironment, strongly supporting the prognostic significance of JAK2 mutation for determining the response to ICI treatment.

We identified the underlying biological processes and pathways between JAK2-mutated patients and JAK2-wild by first performing a differential expression analysis between JAK2 mutant-type and JAK2 wild-type patients and then performed enrichment analyses of functions and pathways for the respective top 100 most significantly highly expressed genes. Functional and pathway enrichment analyses revealed that JAK2-mutated patients exhibited enrichment in “cytokinesis” biological processes (Fig. 3B) and “cytokine-cytokine receptor interaction” and “chemokine signaling pathway” immune-related pathways (Fig. 3D). In contrast, JAK2-wild patients were mainly enriched in “regulation of membrane potential” biological processes (Fig. 3C) and “glutamatergic synapse” non-immune-related pathways (Fig. 3E), indicating that mutated and nonmutated JAK2 act through different mechanisms.

### Potential extrinsic factors affecting the immune response of JAK2-mutated and JAK2-wild cancers

**Fig. 4:**
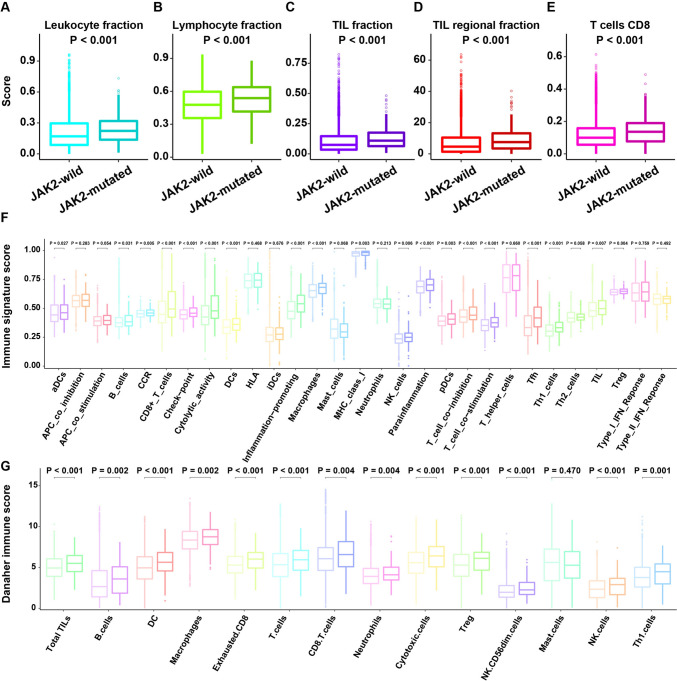
The landscape of immune cell infiltration in JAK2-wild and JAK2-mutated patients. **A** The leukocyte fraction was compared between JAK2-wild and JAK2-mutated patients in TCGA cohort. **B** The lymphocyte fraction was compared between JAK2-wild and JAK2-mutated patients in the validation cohort. **C** The TIL fraction was compared between JAK2-wild and JAK2-mutated patients in TCGA cohort. **D** The TIL regional fraction was compared between JAK2-wild and JAK2-mutated patients in TCGA cohort. **E** The level of CD8^+^ T cells was compared between JAK2-wild and JAK2-mutated patients in TCGA cohort. **F** The immune signature score was compared between JAK2-wild (light color) and JAK2-mutated (dark color) patients in TCGA cohort. **G** The Danaher immune score was compared between JAK2-wild (light color) and JAK2-mutated (dark color) patients in TCGA cohort

Differences in the tumor microenvironment (TME) phenotype cause JAK2-mutated and JAK2-wild cancers to be characterized by different immune response landscapes. Immune cell infiltration into the TME is a prerequisite for anticancer immune activity [33, 34]. First, we compared the white blood cell fraction between JAK2-wild cancers and JAK2-mutated cancers using DNA methylation array data and discovered that JAK2-mutated cancers contained a greater white blood cell fraction (*P* < 0.001, Fig. 4A). Second, we compared the lymphocyte fraction (a vital population of white blood cells) between JAK2-wild cancers and JAK2-mutated cancers with the CIBERSORT approach using RNA-seq data. We detected high lymphocyte fractions in JAK2-mutated cancers (*P* < 0.001, Fig. 4B). Next, we compared the TIL proportion, which was estimated by multiplying the estimated white blood cell proportion by the estimated lymphocyte proportion between JAK2-mutated and JAK2-wild cancers. The TIL fraction of JAK2-wild cancers was greater than that of JAK2-mutated cancers (*P* < 0.001, Fig. 2C). Finally, we employed TIL proportions reported by Saltz et al., who utilized deep learning approaches to predict TIL proportions from H&E-stained sections [26]. The result of the H&E-based estimation of the TIL fraction (*P* < 0.001, Fig. 4D) was consistent with the molecular genome analysis-based estimation of the TIL fraction (Fig. 4C). In particular, we discovered that JAK2-mutated cancers contained a remarkably greater proportion of immunostimulatory cells (CD8 T cells) (*P* < 0.001, Fig. 4E). Hence, we showed that JAK2-mutated cancers have greater immune cell infiltration than JAK2-wild cancers.

We studied the distribution of immune cells between JAK2-wild cancers and JAK2-mutated cancers using alternative approaches for assessing immune cells to confirm the previously mentioned outcomes. The ssGSEA approach was leveraged to assess the immune status of every patient by studying the expression profiles of 29 immune hallmarks [27]. According to the immune signature score estimated using ssGSEA, JAK2-mutated cancers were characterized by more immune cells, such as TILs (*P* = 0.007) and CD8^+^ T cells (*P* < 0.001, Fig. 4F). Moreover, based on the Danaher immune score [35], JAK2-mutated cancers contained more TILs (*P* < 0.001), CD8^+^ T cells (*P* = 0.004), and cytotoxic lymphocytes (*P* < 0.001, Fig. 4G).

**Fig. 5:**
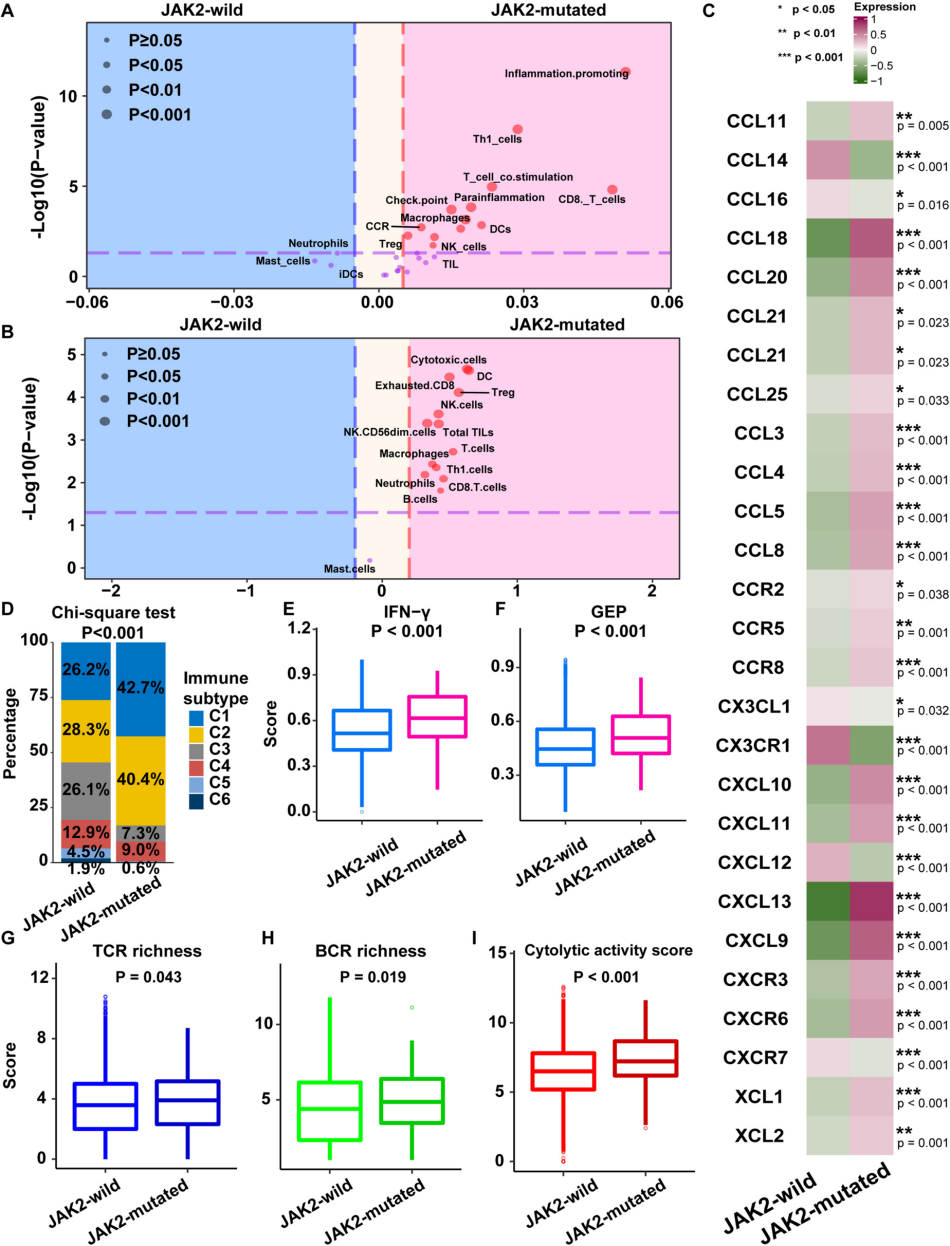
The landscape of immune signatures of JAK2-wild and JAK2-mutated patients. **A** Comparison of immune signature scores between JAK2-wild and JAK2-mutated patients in TCGA cohort. **B** Comparison of Danaher immune scores between JAK2-wild and JAK2-mutated patients in TCGA cohort. **C** Comparison of chemokines between JAK2-wild and JAK2-mutated patients in TCGA cohort. **D** Comparison of immune subtypes between JAK2-wild and JAK2-mutated patients in TCGA cohort. **E** The IFN-γ score was compared between JAK2-wild and JAK2-mutated patients in TCGA cohort. **F** The GEP score was compared between JAK2-wild and JAK2-mutated patients in TCGA cohort. **G** TCR richness was compared between JAK2-wild and JAK2-mutated patients in TCGA cohort. **H** BCR richness was compared between JAK2-wild and JAK2-mutated patients in TCGA cohort. **I** The CYT activity score was compared between JAK2-wild and JAK2-mutated patients in TCGA cohort

In addition, immune signatures (Fig. 5A) and Danaher immune scores (Fig. 5B) were evidently enhanced in JAK2-mutated cancers compared to JAK2-wild cancers. When chemotactic factors bind specific acceptors, immune cells accumulate within the TME [36]. We discovered that JAK2-mutated cancers displayed greater expression of proinflammatory chemotactic factors (Fig. 5C), which have been shown to attract CD8^+^ T cells [37], and their expression is increased when the JAK signaling pathway is activated [38]. The increased chemotactic factor expression in JAK2-mutated cancers was consistent with the finding of greater immunocyte infiltration in JAK2-mutated cancers, revealing that JAK2-wild tumors are capable of attracting immune cells to induce an extrinsic immune response. In addition, Thorsson et al. identified six immune subtypes across cancer types: wound healing (C1), IFN-γdominant (C2), inflammatory (C3), lymphocyte-depleted (C4), immunologically quiet (C5), and TGF-β-dominant (C6) [24]. The IFN-γ dominant subtype has the highest lymphocytic infiltrate, a CD8^+^ T-cell-associated signature, and the highest M1 content. We found that JAK2-mutant patients were mainly enriched in the wound healing (C1) and IFN-γ-dominant immune (C2) subtypes (Fig. 5D). The IFN-γ and the T-cell inflammation-related gene expression profiles (GEPs) were extracted from the study by Ayers [39]. IFN-γ is a critical driver of PD-L1 expression in cancer and host cells [39]. The T-cell inflammation-related GEP can predict the response to ICI therapy in patients with various cancers [39]. We used the GSVA method to evaluate the IFN-γ and GEP scores and found that JAK2-mutated patients had higher expression of IFN-γ and GEP scores than JAK2-wild patients (Fig. 5E, F). Nonsilent somatic mutations in coding regions of genes produce neoantigens that are recognized by T cells with different antigen-specific TCRs [40]. We detected remarkably higher TCR (*P* = 0.043, Fig. 5G) and BCR richness (*P* = 0.019, Fig. 5H) in JAK2-mutated cancers than in JAK2-wild cancers. After antigen-triggered activation, T cells in tumors expand to produce an effector pool that exerts cytotoxic effects. Hence, the CYT score was calculated to explore the interaction between immune system stimulation and cancer. We discovered that JAK2-MUT cancers had remarkably higher CYT scores (*P* < 0.001, Fig. 5I). Based on these results, we concluded that JAK2-mutated cancers contain more immune cells and exhibit greater TCR diversity, enabling them to recognize cancer antigens and induce more potent cancer-eliminating effects.

## Discussion

JAKs play an essential role in the responses to cytokines, hormones, and growth factors [41–43]. JAK–STAT signal transduction regulates the immune system to directly induce the transcription of various genes and translation of proteins associated with cell proliferation and survival, and these processes are specifically involved in cancer cell identification and cancer-driven immune escape [44]. Furthermore, the JAK–STAT pathway regulates the expression levels of PD-L1 and PD-L2 [45], which are presumed to be related to the immunotherapy response. Here, we explored the association between JAK2 (a member of the JAK family) and ICI treatment outcomes in an ICI-treated cohort with meticulously curated outcome and genome data. We found that JAK2 mutation was associated with the response to ICI therapy. We also discovered that JAK2 mutation was a specific and independent prognostic factor for the outcomes of ICI treatment. These results are consistent with that the study by Zhang et al. showing that patients with JAK2-mutated MSI-h colorectal cancer might benefit from anti-PD-1 therapy [46].

A positive relationship between the density of immune cell infiltration and the immune response has been observed in various types of tumors [2]. Therefore, we explored the relationship between JAK2 mutation and immune cell infiltration. In TCGA cohort with multiple omics data, we found that JAK2-mutated tumors had a higher TMB, higher level of CD8 + T-cell infiltration and higher immune score, suggesting that JAK2-mutated tumors have enhanced antitumor immune activity. In addition, overexpression of immune checkpoint factors, such as PD-1 and CTLA4, was detected in JAK2-mutated tumors. Therefore, the better outcome of ICI therapy in patients with JAK2-mutated tumors is likely due to the strong immunogenicity, activated antitumor immunity, and elevated immune checkpoint factor expression in these tumors.

Our research has several innovations. Among the numerous markers associated with the immunotherapy response, high PD-L1 expression and a high TMB have been shown to be associated with a better clinical benefit in patients receiving ICI therapy [2]. Nevertheless, different identification methods and platforms vary widely, both markers are continuous variables, and a uniform cut-off point has not been established to identify which patients will respond to ICI treatment [47, 48]. In comparison, JAK2 mutation can be identified easily using the next-generation sequencing, and its presence was remarkably associated with better ICI treatment outcomes in the present study. In addition, regarding the tumor immune microenvironment, our study revealed that JAK2-mutated cancers had abundant immune cells and greater immune activity. Hence, JAK2 mutation may serve as a potential predictive biomarker to identify patients who may benefit from ICI therapy, which will aid physicians in clinical decision-making.

Through various complex analyses, we identified and validated a biomarker for predicting patients who are suitable for ICI treatment, but we also recognize several limitations. First, this study was limited to retrospective sample verification. The accuracy, reliability, and validity of the results must be confirmed in further prospective clinical trials. As more immunotherapy datasets are published in the future, we will validate the predictive power of JAK2 mutation in these published datasets. At the same time, we are also building our own immunotherapy cohort and validating it in our own cohort. Second, further physiological and pathophysiological experiments are required to confirm the correlations between JAK2 mutation and increased cancer immunogenicity and anticancer immunity, such as in vivo and in vitro experiments to explore the role of JAK2 in ICI treatment. More importantly, the use of anti-PD-L1 drugs to verify the immunotherapy response of JAK2 mutant mice also requires further study. Third, CALR-mutated myeloproliferative neoplasms (MPNs) have been shown to induce a favorable T-cell response to possible ICI therapy [49]. Therefore, our study adds to this concept and possibly adds to the treatment of JAK2-mutated patients. The substitution of valine with phenylalanine at amino acid 617 of the JAK2 gene (JAK2 p.V617F) occurs in a high proportion of patients with MPNs [50]. In the Nangalia study, we found that all 113 JAK2-mutated patients with MPNs carried the p.V617F mutation [50]. The proportion of JAK2 p.V617F mutation was as high as 72.6%. However, we did not detect the JAK2 p.V617F mutation in our training cohort, probably because patients with MPN tumors were not included in the training cohort. Therefore, a disadvantage of our study is that it does not cover as many tumor types as MPNs. In the future, as an increasing number of ICI treatment datasets are published, corresponding studies on whether the JAK2 p.V617F mutation can be used as a tumor antigen for immunotherapy and will also need to be conducted.

## Conclusions

In summary, this study is the first to support the hypothesis that JAK2-mutated patients exhibit increased cancer immunogenicity and anticancer immunity, leading to an enhanced response to and long-term survival benefits of ICI therapy. JAK2 mutation might serve as a predictive biological marker for identifying appropriate patients for ICI treatment.

### Supplementary Information

Below is the link to the electronic supplementary material.

Supplementary file 1 (DOCX 72 KB)

Fig. S1. Process used to screen patients.(A) Overall workflow describing the process used to screen patients in the discovery cohort treated with ICI therapy. (B) Overall workflow describing the process used to screen in the verification cohort treated with ICI therapy. (C) Overall workflow describing the process for screening patients in the non-ICI cohort from the study by Zehir et al. Supplementary file 2 (TIF 1129 KB)

Fig. S2. Overall survival of JAK2-wild and JAK2-mutated patients.(A) Kaplan-Meier curves of OS were compared between JAK2-wild and JAK2-mutated patients in the validation cohort. (B) Kaplan-Meier curves of OS were compared between JAK2-wild and JAK2-mutated patients in the non-ICI-treated cohort.Supplementary file 2 (TIF 2171 KB)

## Data Availability

All data generated or analyzed during this study are obtained after processing were acquired from cBioPortal (https://www.cbioportal.org) and previous articles.
