# Supplementary material for: Identification and validation of the association of Janus kinase 2 mutations with the response to immune checkpoint inhibitor therapy
Source: Inflamm Res. 2024 Jan 10;73(2):263–76. doi: 10.1007/s00011-023-01833-w (PMC10824873; doi:10.1007/s00011-023-01833-w)
Supplement: Supplementary file 1 — (DOCX 72 KB) [file 11_2023_1833_MOESM1_ESM.docx]

**Table S1. Survival analysis was performed with 16 JAK2-MUT patients and 16 JAK2-WT patients randomly selected from 586 JAK2-WT patients, with 1,000 samplings.**

| **Seed** | **The total number of JAK2-WT patients** | **The total number of JAK2-MUT patients** | **Median PFS of JAK2-WT patients** | **Median PFS of JAK2-MUT patients** | **P value**  **(log-rank test)** | **Hazard ratio** |
| --- | --- | --- | --- | --- | --- | --- |
| 1 | 16 | 16 | 4.380 | 38.067 | 0.068 | 0.446 |
| 2 | 16 | 16 | 5.825 | 38.067 | 0.093 | 0.475 |
| 3 | 16 | 16 | 2.650 | 38.067 | 0.008 | 0.291 |
| 4 | 16 | 16 | 3.880 | 38.067 | 0.030 | 0.370 |
| 5 | 16 | 16 | 2.665 | 38.067 | 0.030 | 0.386 |
| 6 | 16 | 16 | 2.433 | 38.067 | 0.004 | 0.271 |
| 7 | 16 | 16 | 2.570 | 38.067 | 0.001 | 0.222 |
| 8 | 16 | 16 | 7.170 | 38.067 | 0.120 | 0.486 |
| 9 | 16 | 16 | 2.765 | 38.067 | 0.020 | 0.370 |
| 10 | 16 | 16 | 2.800 | 38.067 | 0.010 | 0.309 |
| 11 | 16 | 16 | 2.567 | 38.067 | 0.000 | 0.195 |
| 12 | 16 | 16 | 44.233 | 38.067 | 0.957 | 1.028 |
| 13 | 16 | 16 | 2.518 | 38.067 | 0.006 | 0.295 |
| 14 | 16 | 16 | 5.400 | 38.067 | 0.005 | 0.279 |
| 15 | 16 | 16 | 4.352 | 38.067 | 0.092 | 0.460 |
| 16 | 16 | 16 | 6.380 | 38.067 | 0.027 | 0.355 |
| 17 | 16 | 16 | 3.025 | 38.067 | 0.016 | 0.332 |
| 18 | 16 | 16 | 5.100 | 38.067 | 0.011 | 0.319 |
| 19 | 16 | 16 | 3.560 | 38.067 | 0.014 | 0.332 |
| 20 | 16 | 16 | 3.317 | 38.067 | 0.054 | 0.419 |
| 21 | 16 | 16 | 2.760 | 38.067 | 0.018 | 0.323 |
| 22 | 16 | 16 | 2.967 | 38.067 | 0.031 | 0.377 |
| 23 | 16 | 16 | 3.500 | 38.067 | 0.058 | 0.421 |
| 24 | 16 | 16 | 2.867 | 38.067 | 0.003 | 0.268 |
| 25 | 16 | 16 | 3.200 | 38.067 | 0.028 | 0.360 |
| 26 | 16 | 16 | 4.300 | 38.067 | 0.048 | 0.393 |
| 27 | 16 | 16 | 3.033 | 38.067 | 0.054 | 0.412 |
| 28 | 16 | 16 | 4.800 | 38.067 | 0.023 | 0.354 |
| 29 | 16 | 16 | 3.100 | 38.067 | 0.073 | 0.418 |
| 30 | 16 | 16 | 5.633 | 38.067 | 0.118 | 0.481 |
| 31 | 16 | 16 | 6.033 | 38.067 | 0.255 | 0.575 |
| 32 | 16 | 16 | 5.590 | 38.067 | 0.243 | 0.577 |
| 33 | 16 | 16 | 5.185 | 38.067 | 0.091 | 0.450 |
| 34 | 16 | 16 | 5.750 | 38.067 | 0.044 | 0.397 |
| 35 | 16 | 16 | 4.700 | 38.067 | 0.184 | 0.530 |
| 36 | 16 | 16 | 4.677 | 38.067 | 0.161 | 0.525 |
| 37 | 16 | 16 | 5.400 | 38.067 | 0.345 | 0.640 |
| 38 | 16 | 16 | 2.733 | 38.067 | 0.006 | 0.288 |
| 39 | 16 | 16 | 3.850 | 38.067 | 0.102 | 0.480 |
| 40 | 16 | 16 | 8.290 | 38.067 | 0.241 | 0.564 |
| 41 | 16 | 16 | 2.833 | 38.067 | 0.005 | 0.288 |
| 42 | 16 | 16 | 4.133 | 38.067 | 0.232 | 0.568 |
| 43 | 16 | 16 | 2.800 | 38.067 | 0.003 | 0.257 |
| 44 | 16 | 16 | 4.760 | 38.067 | 0.097 | 0.475 |
| 45 | 16 | 16 | 6.585 | 38.067 | 0.144 | 0.505 |
| 46 | 16 | 16 | 5.990 | 38.067 | 0.087 | 0.442 |
| 47 | 16 | 16 | 5.470 | 38.067 | 0.070 | 0.416 |
| 48 | 16 | 16 | 2.813 | 38.067 | 0.002 | 0.249 |
| 49 | 16 | 16 | 3.438 | 38.067 | 0.054 | 0.429 |
| 50 | 16 | 16 | 15.760 | 38.067 | 0.320 | 0.615 |
| 51 | 16 | 16 | 3.500 | 38.067 | 0.006 | 0.279 |
| 52 | 16 | 16 | 3.220 | 38.067 | 0.046 | 0.415 |
| 53 | 16 | 16 | 3.132 | 38.067 | 0.125 | 0.475 |
| 54 | 16 | 16 | 2.550 | 38.067 | 0.024 | 0.359 |
| 55 | 16 | 16 | 7.295 | 38.067 | 0.279 | 0.607 |
| 56 | 16 | 16 | 7.900 | 38.067 | 0.479 | 0.698 |
| 57 | 16 | 16 | 5.400 | 38.067 | 0.051 | 0.404 |
| 58 | 16 | 16 | 2.883 | 38.067 | 0.158 | 0.523 |
| 59 | 16 | 16 | 4.740 | 38.067 | 0.041 | 0.395 |
| 60 | 16 | 16 | 2.320 | 38.067 | 0.001 | 0.244 |
| 61 | 16 | 16 | 3.500 | 38.067 | 0.035 | 0.386 |
| 62 | 16 | 16 | 4.270 | 38.067 | 0.069 | 0.421 |
| 63 | 16 | 16 | 5.330 | 38.067 | 0.067 | 0.447 |
| 64 | 16 | 16 | 2.240 | 38.067 | 0.001 | 0.233 |
| 65 | 16 | 16 | 3.570 | 38.067 | 0.138 | 0.506 |
| 66 | 16 | 16 | 3.870 | 38.067 | 0.040 | 0.401 |
| 67 | 16 | 16 | 5.433 | 38.067 | 0.030 | 0.367 |
| 68 | 16 | 16 | 3.825 | 38.067 | 0.158 | 0.517 |
| 69 | 16 | 16 | 7.817 | 38.067 | 0.373 | 0.644 |
| 70 | 16 | 16 | 3.220 | 38.067 | 0.060 | 0.407 |
| 71 | 16 | 16 | 4.140 | 38.067 | 0.113 | 0.488 |
| 72 | 16 | 16 | 3.085 | 38.067 | 0.001 | 0.222 |
| 73 | 16 | 16 | 3.340 | 38.067 | 0.007 | 0.305 |
| 74 | 16 | 16 | 2.760 | 38.067 | 0.000 | 0.177 |
| 75 | 16 | 16 | 4.630 | 38.067 | 0.186 | 0.545 |
| 76 | 16 | 16 | 5.100 | 38.067 | 0.111 | 0.464 |
| 77 | 16 | 16 | 6.253 | 38.067 | 0.661 | 0.808 |
| 78 | 16 | 16 | 2.585 | 38.067 | 0.019 | 0.366 |
| 79 | 16 | 16 | 2.485 | 38.067 | 0.000 | 0.220 |
| 80 | 16 | 16 | 2.502 | 38.067 | 0.002 | 0.251 |
| 81 | 16 | 16 | 6.170 | 38.067 | 0.170 | 0.529 |
| 82 | 16 | 16 | 3.260 | 38.067 | 0.015 | 0.319 |
| 83 | 16 | 16 | 3.185 | 38.067 | 0.055 | 0.403 |
| 84 | 16 | 16 | 3.500 | 38.067 | 0.241 | 0.562 |
| 85 | 16 | 16 | 8.290 | 38.067 | 0.319 | 0.631 |
| 86 | 16 | 16 | 5.370 | 38.067 | 0.033 | 0.397 |
| 87 | 16 | 16 | 3.175 | 38.067 | 0.008 | 0.268 |
| 88 | 16 | 16 | 5.130 | 38.067 | 0.160 | 0.496 |
| 89 | 16 | 16 | 4.630 | 38.067 | 0.072 | 0.434 |
| 90 | 16 | 16 | 2.760 | 38.067 | 0.007 | 0.292 |
| 91 | 16 | 16 | 2.910 | 38.067 | 0.006 | 0.283 |
| 92 | 16 | 16 | 5.185 | 38.067 | 0.082 | 0.442 |
| 93 | 16 | 16 | 8.000 | 38.067 | 0.089 | 0.458 |
| 94 | 16 | 16 | 2.948 | 38.067 | 0.018 | 0.342 |
| 95 | 16 | 16 | 4.117 | 38.067 | 0.120 | 0.478 |
| 96 | 16 | 16 | 2.430 | 38.067 | 0.001 | 0.243 |
| 97 | 16 | 16 | 3.967 | 38.067 | 0.010 | 0.311 |
| 98 | 16 | 16 | 2.760 | 38.067 | 0.009 | 0.290 |
| 99 | 16 | 16 | 3.500 | 38.067 | 0.038 | 0.386 |
| 100 | 16 | 16 | 4.500 | 38.067 | 0.133 | 0.489 |
| 101 | 16 | 16 | 3.050 | 38.067 | 0.050 | 0.401 |
| 102 | 16 | 16 | 6.280 | 38.067 | 0.368 | 0.636 |
| 103 | 16 | 16 | 3.305 | 38.067 | 0.002 | 0.253 |
| 104 | 16 | 16 | 3.283 | 38.067 | 0.009 | 0.312 |
| 105 | 16 | 16 | 2.270 | 38.067 | 0.001 | 0.239 |
| 106 | 16 | 16 | 4.000 | 38.067 | 0.024 | 0.362 |
| 107 | 16 | 16 | 4.220 | 38.067 | 0.106 | 0.469 |
| 108 | 16 | 16 | 3.083 | 38.067 | 0.065 | 0.424 |
| 109 | 16 | 16 | 2.140 | 38.067 | 0.001 | 0.225 |
| 110 | 16 | 16 | 32.860 | 38.067 | 0.602 | 0.780 |
| 111 | 16 | 16 | 2.800 | 38.067 | 0.043 | 0.393 |
| 112 | 16 | 16 | 4.800 | 38.067 | 0.083 | 0.446 |
| 113 | 16 | 16 | 3.325 | 38.067 | 0.103 | 0.470 |
| 114 | 16 | 16 | 10.630 | 38.067 | 0.535 | 0.724 |
| 115 | 16 | 16 | 2.533 | 38.067 | 0.001 | 0.231 |
| 116 | 16 | 16 | 3.133 | 38.067 | 0.002 | 0.212 |
| 117 | 16 | 16 | 3.110 | 38.067 | 0.004 | 0.272 |
| 118 | 16 | 16 | 4.140 | 38.067 | 0.016 | 0.336 |
| 119 | 16 | 16 | 3.100 | 38.067 | 0.007 | 0.290 |
| 120 | 16 | 16 | 2.315 | 38.067 | 0.002 | 0.264 |
| 121 | 16 | 16 | 3.150 | 38.067 | 0.194 | 0.531 |
| 122 | 16 | 16 | 4.400 | 38.067 | 0.165 | 0.540 |
| 123 | 16 | 16 | 6.120 | 38.067 | 0.086 | 0.446 |
| 124 | 16 | 16 | 2.760 | 38.067 | 0.009 | 0.307 |
| 125 | 16 | 16 | 6.350 | 38.067 | 0.272 | 0.600 |
| 126 | 16 | 16 | 5.833 | 38.067 | 0.068 | 0.434 |
| 127 | 16 | 16 | 5.990 | 38.067 | 0.076 | 0.436 |
| 128 | 16 | 16 | 4.060 | 38.067 | 0.067 | 0.424 |
| 129 | 16 | 16 | 7.900 | 38.067 | 0.548 | 0.746 |
| 130 | 16 | 16 | 2.680 | 38.067 | 0.016 | 0.336 |
| 131 | 16 | 16 | 3.585 | 38.067 | 0.012 | 0.271 |
| 132 | 16 | 16 | 4.435 | 38.067 | 0.012 | 0.318 |
| 133 | 16 | 16 | 3.230 | 38.067 | 0.170 | 0.511 |
| 134 | 16 | 16 | 3.950 | 38.067 | 0.332 | 0.619 |
| 135 | 16 | 16 | 3.490 | 38.067 | 0.086 | 0.447 |
| 136 | 16 | 16 | 3.280 | 38.067 | 0.003 | 0.270 |
| 137 | 16 | 16 | 2.533 | 38.067 | 0.000 | 0.199 |
| 138 | 16 | 16 | 2.667 | 38.067 | 0.009 | 0.306 |
| 139 | 16 | 16 | 3.500 | 38.067 | 0.043 | 0.389 |
| 140 | 16 | 16 | 2.833 | 38.067 | 0.042 | 0.396 |
| 141 | 16 | 16 | 2.963 | 38.067 | 0.007 | 0.296 |
| 142 | 16 | 16 | 8.402 | 38.067 | 0.140 | 0.512 |
| 143 | 16 | 16 | 5.400 | 38.067 | 0.340 | 0.629 |
| 144 | 16 | 16 | 6.130 | 38.067 | 0.059 | 0.417 |
| 145 | 16 | 16 | 6.120 | 38.067 | 0.408 | 0.669 |
| 146 | 16 | 16 | 3.455 | 38.067 | 0.089 | 0.472 |
| 147 | 16 | 16 | 3.380 | 38.067 | 0.064 | 0.417 |
| 148 | 16 | 16 | 2.270 | 38.067 | 0.006 | 0.296 |
| 149 | 16 | 16 | 5.040 | 38.067 | 0.191 | 0.547 |
| 150 | 16 | 16 | 2.760 | 38.067 | 0.030 | 0.377 |
| 151 | 16 | 16 | 3.770 | 38.067 | 0.560 | 0.745 |
| 152 | 16 | 16 | 2.800 | 38.067 | 0.023 | 0.370 |
| 153 | 16 | 16 | 2.220 | 38.067 | 0.002 | 0.262 |
| 154 | 16 | 16 | 3.680 | 38.067 | 0.076 | 0.430 |
| 155 | 16 | 16 | 8.290 | 38.067 | 0.291 | 0.587 |
| 156 | 16 | 16 | 40.933 | 38.067 | 0.650 | 1.267 |
| 157 | 16 | 16 | 4.700 | 38.067 | 0.276 | 0.586 |
| 158 | 16 | 16 | 4.270 | 38.067 | 0.142 | 0.470 |
| 159 | 16 | 16 | 5.400 | 38.067 | 0.054 | 0.429 |
| 160 | 16 | 16 | 10.633 | 38.067 | 0.315 | 0.620 |
| 161 | 16 | 16 | 4.965 | 38.067 | 0.105 | 0.462 |
| 162 | 16 | 16 | 5.430 | 38.067 | 0.198 | 0.534 |
| 163 | 16 | 16 | 8.290 | 38.067 | 0.316 | 0.617 |
| 164 | 16 | 16 | 4.125 | 38.067 | 0.036 | 0.384 |
| 165 | 16 | 16 | 3.605 | 38.067 | 0.064 | 0.443 |
| 166 | 16 | 16 | 4.410 | 38.067 | 0.105 | 0.464 |
| 167 | 16 | 16 | 4.140 | 38.067 | 0.217 | 0.541 |
| 168 | 16 | 16 | 4.370 | 38.067 | 0.200 | 0.553 |
| 169 | 16 | 16 | 3.390 | 38.067 | 0.043 | 0.406 |
| 170 | 16 | 16 | 4.140 | 38.067 | 0.086 | 0.440 |
| 171 | 16 | 16 | 4.200 | 38.067 | 0.061 | 0.416 |
| 172 | 16 | 16 | 2.760 | 38.067 | 0.005 | 0.259 |
| 173 | 16 | 16 | 3.620 | 38.067 | 0.019 | 0.339 |
| 174 | 16 | 16 | 5.400 | 38.067 | 0.146 | 0.491 |
| 175 | 16 | 16 | 2.567 | 38.067 | 0.130 | 0.470 |
| 176 | 16 | 16 | 5.130 | 38.067 | 0.015 | 0.328 |
| 177 | 16 | 16 | 4.110 | 38.067 | 0.031 | 0.375 |
| 178 | 16 | 16 | 4.690 | 38.067 | 0.078 | 0.448 |
| 179 | 16 | 16 | 3.885 | 38.067 | 0.038 | 0.387 |
| 180 | 16 | 16 | 3.500 | 38.067 | 0.030 | 0.370 |
| 181 | 16 | 16 | 3.090 | 38.067 | 0.115 | 0.474 |
| 182 | 16 | 16 | 3.565 | 38.067 | 0.012 | 0.327 |
| 183 | 16 | 16 | 3.075 | 38.067 | 0.013 | 0.348 |
| 184 | 16 | 16 | 2.650 | 38.067 | 0.001 | 0.263 |
| 185 | 16 | 16 | 5.590 | 38.067 | 0.014 | 0.335 |
| 186 | 16 | 16 | 4.970 | 38.067 | 0.117 | 0.471 |
| 187 | 16 | 16 | 3.265 | 38.067 | 0.011 | 0.320 |
| 188 | 16 | 16 | 5.350 | 38.067 | 0.199 | 0.553 |
| 189 | 16 | 16 | 5.095 | 38.067 | 0.013 | 0.328 |
| 190 | 16 | 16 | 2.967 | 38.067 | 0.035 | 0.393 |
| 191 | 16 | 16 | 5.570 | 38.067 | 0.167 | 0.522 |
| 192 | 16 | 16 | 3.368 | 38.067 | 0.055 | 0.410 |
| 193 | 16 | 16 | 3.245 | 38.067 | 0.041 | 0.389 |
| 194 | 16 | 16 | 4.540 | 38.067 | 0.063 | 0.409 |
| 195 | 16 | 16 | 5.302 | 38.067 | 0.188 | 0.549 |
| 196 | 16 | 16 | 4.215 | 38.067 | 0.120 | 0.477 |
| 197 | 16 | 16 | 5.500 | 38.067 | 0.255 | 0.571 |
| 198 | 16 | 16 | 2.370 | 38.067 | 0.109 | 0.470 |
| 199 | 16 | 16 | 10.400 | 38.067 | 0.135 | 0.483 |
| 200 | 16 | 16 | 2.100 | 38.067 | 0.007 | 0.301 |
| 201 | 16 | 16 | 5.570 | 38.067 | 0.505 | 0.729 |
| 202 | 16 | 16 | 3.915 | 38.067 | 0.116 | 0.473 |
| 203 | 16 | 16 | 3.375 | 38.067 | 0.054 | 0.409 |
| 204 | 16 | 16 | 4.800 | 38.067 | 0.328 | 0.629 |
| 205 | 16 | 16 | 3.890 | 38.067 | 0.071 | 0.453 |
| 206 | 16 | 16 | 2.400 | 38.067 | 0.002 | 0.254 |
| 207 | 16 | 16 | 3.800 | 38.067 | 0.002 | 0.229 |
| 208 | 16 | 16 | 2.320 | 38.067 | 0.004 | 0.278 |
| 209 | 16 | 16 | 3.100 | 38.067 | 0.009 | 0.297 |
| 210 | 16 | 16 | 3.420 | 38.067 | 0.076 | 0.450 |
| 211 | 16 | 16 | 4.515 | 38.067 | 0.024 | 0.357 |
| 212 | 16 | 16 | 3.380 | 38.067 | 0.129 | 0.505 |
| 213 | 16 | 16 | 3.500 | 38.067 | 0.063 | 0.410 |
| 214 | 16 | 16 | 3.050 | 38.067 | 0.100 | 0.470 |
| 215 | 16 | 16 | 4.170 | 38.067 | 0.007 | 0.287 |
| 216 | 16 | 16 | 4.510 | 38.067 | 0.077 | 0.415 |
| 217 | 16 | 16 | 5.202 | 38.067 | 0.182 | 0.522 |
| 218 | 16 | 16 | 3.225 | 38.067 | 0.030 | 0.371 |
| 219 | 16 | 16 | 4.332 | 38.067 | 0.087 | 0.471 |
| 220 | 16 | 16 | 5.020 | 38.067 | 0.029 | 0.347 |
| 221 | 16 | 16 | 4.800 | 38.067 | 0.150 | 0.518 |
| 222 | 16 | 16 | 5.470 | 38.067 | 0.004 | 0.263 |
| 223 | 16 | 16 | 2.518 | 38.067 | 0.001 | 0.222 |
| 224 | 16 | 16 | 2.915 | 38.067 | 0.056 | 0.412 |
| 225 | 16 | 16 | 1.935 | 38.067 | 0.001 | 0.195 |
| 226 | 16 | 16 | 2.800 | 38.067 | 0.014 | 0.330 |
| 227 | 16 | 16 | 4.378 | 38.067 | 0.042 | 0.391 |
| 228 | 16 | 16 | 3.017 | 38.067 | 0.134 | 0.508 |
| 229 | 16 | 16 | 4.240 | 38.067 | 0.035 | 0.369 |
| 230 | 16 | 16 | 3.255 | 38.067 | 0.015 | 0.332 |
| 231 | 16 | 16 | 5.080 | 38.067 | 0.047 | 0.414 |
| 232 | 16 | 16 | 2.900 | 38.067 | 0.045 | 0.385 |
| 233 | 16 | 16 | 3.600 | 38.067 | 0.007 | 0.280 |
| 234 | 16 | 16 | 5.530 | 38.067 | 0.021 | 0.352 |
| 235 | 16 | 16 | 3.133 | 38.067 | 0.008 | 0.306 |
| 236 | 16 | 16 | 2.335 | 38.067 | 0.025 | 0.376 |
| 237 | 16 | 16 | 10.400 | 38.067 | 0.086 | 0.440 |
| 238 | 16 | 16 | 2.800 | 38.067 | 0.003 | 0.253 |
| 239 | 16 | 16 | 6.460 | 38.067 | 0.216 | 0.566 |
| 240 | 16 | 16 | 3.600 | 38.067 | 0.201 | 0.561 |
| 241 | 16 | 16 | 2.665 | 38.067 | 0.037 | 0.401 |
| 242 | 16 | 16 | 4.015 | 38.067 | 0.071 | 0.429 |
| 243 | 16 | 16 | 2.947 | 38.067 | 0.004 | 0.265 |
| 244 | 16 | 16 | 2.660 | 38.067 | 0.006 | 0.289 |
| 245 | 16 | 16 | 2.747 | 38.067 | 0.005 | 0.289 |
| 246 | 16 | 16 | 3.980 | 38.067 | 0.018 | 0.320 |
| 247 | 16 | 16 | 6.733 | 38.067 | 0.038 | 0.378 |
| 248 | 16 | 16 | 6.080 | 38.067 | 0.141 | 0.494 |
| 249 | 16 | 16 | 3.117 | 38.067 | 0.008 | 0.303 |
| 250 | 16 | 16 | 5.400 | 38.067 | 0.060 | 0.426 |
| 251 | 16 | 16 | 6.130 | 38.067 | 0.266 | 0.571 |
| 252 | 16 | 16 | 2.685 | 38.067 | 0.004 | 0.280 |
| 253 | 16 | 16 | 2.233 | 38.067 | 0.034 | 0.396 |
| 254 | 16 | 16 | 2.570 | 38.067 | 0.080 | 0.445 |
| 255 | 16 | 16 | 4.085 | 38.067 | 0.019 | 0.344 |
| 256 | 16 | 16 | 4.715 | 38.067 | 0.032 | 0.374 |
| 257 | 16 | 16 | 2.383 | 38.067 | 0.009 | 0.326 |
| 258 | 16 | 16 | 4.030 | 38.067 | 0.065 | 0.448 |
| 259 | 16 | 16 | 4.440 | 38.067 | 0.011 | 0.326 |
| 260 | 16 | 16 | 3.390 | 38.067 | 0.027 | 0.365 |
| 261 | 16 | 16 | 5.525 | 38.067 | 0.284 | 0.600 |
| 262 | 16 | 16 | 5.870 | 38.067 | 0.391 | 0.649 |
| 263 | 16 | 16 | 2.500 | 38.067 | 0.007 | 0.318 |
| 264 | 16 | 16 | 4.085 | 38.067 | 0.075 | 0.434 |
| 265 | 16 | 16 | 2.750 | 38.067 | 0.013 | 0.329 |
| 266 | 16 | 16 | NA | 38.067 | 0.989 | 0.993 |
| 267 | 16 | 16 | 3.910 | 38.067 | 0.028 | 0.353 |
| 268 | 16 | 16 | 3.820 | 38.067 | 0.030 | 0.368 |
| 269 | 16 | 16 | 4.540 | 38.067 | 0.330 | 0.631 |
| 270 | 16 | 16 | 5.100 | 38.067 | 0.004 | 0.268 |
| 271 | 16 | 16 | 2.385 | 38.067 | 0.124 | 0.495 |
| 272 | 16 | 16 | 4.490 | 38.067 | 0.055 | 0.424 |
| 273 | 16 | 16 | 4.800 | 38.067 | 0.037 | 0.376 |
| 274 | 16 | 16 | 4.000 | 38.067 | 0.170 | 0.519 |
| 275 | 16 | 16 | 3.935 | 38.067 | 0.017 | 0.343 |
| 276 | 16 | 16 | 3.165 | 38.067 | 0.009 | 0.303 |
| 277 | 16 | 16 | 5.902 | 38.067 | 0.207 | 0.552 |
| 278 | 16 | 16 | 3.723 | 38.067 | 0.134 | 0.491 |
| 279 | 16 | 16 | 3.625 | 38.067 | 0.028 | 0.369 |
| 280 | 16 | 16 | 9.200 | 38.067 | 0.381 | 0.644 |
| 281 | 16 | 16 | 2.730 | 38.067 | 0.022 | 0.346 |
| 282 | 16 | 16 | 10.630 | 38.067 | 0.896 | 0.937 |
| 283 | 16 | 16 | 5.732 | 38.067 | 0.054 | 0.428 |
| 284 | 16 | 16 | 2.783 | 38.067 | 0.000 | 0.191 |
| 285 | 16 | 16 | 3.472 | 38.067 | 0.062 | 0.406 |
| 286 | 16 | 16 | 2.767 | 38.067 | 0.006 | 0.295 |
| 287 | 16 | 16 | 3.147 | 38.067 | 0.005 | 0.287 |
| 288 | 16 | 16 | 3.800 | 38.067 | 0.050 | 0.390 |
| 289 | 16 | 16 | 2.730 | 38.067 | 0.002 | 0.254 |
| 290 | 16 | 16 | 4.330 | 38.067 | 0.146 | 0.523 |
| 291 | 16 | 16 | 2.865 | 38.067 | 0.005 | 0.276 |
| 292 | 16 | 16 | 3.700 | 38.067 | 0.064 | 0.412 |
| 293 | 16 | 16 | 7.350 | 38.067 | 0.345 | 0.641 |
| 294 | 16 | 16 | 2.370 | 38.067 | 0.003 | 0.257 |
| 295 | 16 | 16 | 12.070 | 38.067 | 0.447 | 0.664 |
| 296 | 16 | 16 | 3.500 | 38.067 | 0.026 | 0.355 |
| 297 | 16 | 16 | 8.330 | 38.067 | 0.348 | 0.638 |
| 298 | 16 | 16 | 9.600 | 38.067 | 0.193 | 0.556 |
| 299 | 16 | 16 | 2.763 | 38.067 | 0.125 | 0.494 |
| 300 | 16 | 16 | 2.570 | 38.067 | 0.142 | 0.477 |
| 301 | 16 | 16 | 11.217 | 38.067 | 0.475 | 0.713 |
| 302 | 16 | 16 | 4.050 | 38.067 | 0.054 | 0.410 |
| 303 | 16 | 16 | 4.330 | 38.067 | 0.106 | 0.482 |
| 304 | 16 | 16 | 4.110 | 38.067 | 0.054 | 0.407 |
| 305 | 16 | 16 | 4.133 | 38.067 | 0.101 | 0.479 |
| 306 | 16 | 16 | 9.100 | 38.067 | 0.487 | 0.705 |
| 307 | 16 | 16 | 5.385 | 38.067 | 0.209 | 0.565 |
| 308 | 16 | 16 | 4.000 | 38.067 | 0.096 | 0.448 |
| 309 | 16 | 16 | 8.300 | 38.067 | 0.428 | 0.692 |
| 310 | 16 | 16 | 13.320 | 38.067 | 0.193 | 0.535 |
| 311 | 16 | 16 | 5.770 | 38.067 | 0.357 | 0.632 |
| 312 | 16 | 16 | 4.092 | 38.067 | 0.112 | 0.466 |
| 313 | 16 | 16 | 2.997 | 38.067 | 0.009 | 0.308 |
| 314 | 16 | 16 | 3.820 | 38.067 | 0.034 | 0.377 |
| 315 | 16 | 16 | 2.833 | 38.067 | 0.023 | 0.350 |
| 316 | 16 | 16 | 4.730 | 38.067 | 0.220 | 0.555 |
| 317 | 16 | 16 | 8.190 | 38.067 | 0.341 | 0.633 |
| 318 | 16 | 16 | 2.470 | 38.067 | 0.003 | 0.258 |
| 319 | 16 | 16 | 8.300 | 38.067 | 0.370 | 0.634 |
| 320 | 16 | 16 | 4.140 | 38.067 | 0.060 | 0.411 |
| 321 | 16 | 16 | 4.133 | 38.067 | 0.366 | 0.642 |
| 322 | 16 | 16 | 7.660 | 38.067 | 0.352 | 0.643 |
| 323 | 16 | 16 | 3.600 | 38.067 | 0.021 | 0.338 |
| 324 | 16 | 16 | 2.652 | 38.067 | 0.001 | 0.225 |
| 325 | 16 | 16 | 4.120 | 38.067 | 0.008 | 0.296 |
| 326 | 16 | 16 | 8.290 | 38.067 | 0.334 | 0.623 |
| 327 | 16 | 16 | 2.085 | 38.067 | 0.000 | 0.142 |
| 328 | 16 | 16 | 2.760 | 38.067 | 0.033 | 0.398 |
| 329 | 16 | 16 | 3.910 | 38.067 | 0.011 | 0.318 |
| 330 | 16 | 16 | 4.330 | 38.067 | 0.108 | 0.486 |
| 331 | 16 | 16 | 8.090 | 38.067 | 0.102 | 0.466 |
| 332 | 16 | 16 | 8.000 | 38.067 | 0.255 | 0.583 |
| 333 | 16 | 16 | 2.833 | 38.067 | 0.003 | 0.222 |
| 334 | 16 | 16 | 3.360 | 38.067 | 0.016 | 0.343 |
| 335 | 16 | 16 | 2.600 | 38.067 | 0.050 | 0.416 |
| 336 | 16 | 16 | 4.730 | 38.067 | 0.023 | 0.358 |
| 337 | 16 | 16 | 2.960 | 38.067 | 0.009 | 0.299 |
| 338 | 16 | 16 | 5.650 | 38.067 | 0.209 | 0.561 |
| 339 | 16 | 16 | 6.785 | 38.067 | 0.074 | 0.445 |
| 340 | 16 | 16 | 3.767 | 38.067 | 0.009 | 0.329 |
| 341 | 16 | 16 | 3.112 | 38.067 | 0.011 | 0.320 |
| 342 | 16 | 16 | 2.215 | 38.067 | 0.022 | 0.349 |
| 343 | 16 | 16 | 4.217 | 38.067 | 0.010 | 0.303 |
| 344 | 16 | 16 | 2.468 | 38.067 | 0.002 | 0.251 |
| 345 | 16 | 16 | 3.855 | 38.067 | 0.179 | 0.526 |
| 346 | 16 | 16 | 2.645 | 38.067 | 0.006 | 0.277 |
| 347 | 16 | 16 | 2.785 | 38.067 | 0.009 | 0.320 |
| 348 | 16 | 16 | 4.885 | 38.067 | 0.069 | 0.448 |
| 349 | 16 | 16 | 4.730 | 38.067 | 0.169 | 0.506 |
| 350 | 16 | 16 | 9.200 | 38.067 | 0.452 | 0.693 |
| 351 | 16 | 16 | 3.150 | 38.067 | 0.008 | 0.299 |
| 352 | 16 | 16 | 2.665 | 38.067 | 0.010 | 0.312 |
| 353 | 16 | 16 | 13.750 | 38.067 | 0.237 | 0.558 |
| 354 | 16 | 16 | 5.130 | 38.067 | 0.165 | 0.505 |
| 355 | 16 | 16 | 5.415 | 38.067 | 0.184 | 0.548 |
| 356 | 16 | 16 | 2.730 | 38.067 | 0.080 | 0.448 |
| 357 | 16 | 16 | 6.300 | 38.067 | 0.151 | 0.503 |
| 358 | 16 | 16 | 3.267 | 38.067 | 0.013 | 0.309 |
| 359 | 16 | 16 | 2.865 | 38.067 | 0.008 | 0.294 |
| 360 | 16 | 16 | 4.140 | 38.067 | 0.158 | 0.517 |
| 361 | 16 | 16 | 3.520 | 38.067 | 0.032 | 0.358 |
| 362 | 16 | 16 | 3.830 | 38.067 | 0.174 | 0.515 |
| 363 | 16 | 16 | 2.930 | 38.067 | 0.018 | 0.350 |
| 364 | 16 | 16 | 3.800 | 38.067 | 0.121 | 0.480 |
| 365 | 16 | 16 | 3.277 | 38.067 | 0.014 | 0.329 |
| 366 | 16 | 16 | 3.750 | 38.067 | 0.074 | 0.434 |
| 367 | 16 | 16 | 9.333 | 38.067 | 0.199 | 0.533 |
| 368 | 16 | 16 | 3.220 | 38.067 | 0.012 | 0.318 |
| 369 | 16 | 16 | 4.970 | 38.067 | 0.103 | 0.458 |
| 370 | 16 | 16 | 8.670 | 38.067 | 0.039 | 0.380 |
| 371 | 16 | 16 | 3.705 | 38.067 | 0.038 | 0.385 |
| 372 | 16 | 16 | 2.570 | 38.067 | 0.007 | 0.302 |
| 373 | 16 | 16 | 4.870 | 38.067 | 0.108 | 0.492 |
| 374 | 16 | 16 | 5.370 | 38.067 | 0.125 | 0.501 |
| 375 | 16 | 16 | 5.012 | 38.067 | 0.123 | 0.480 |
| 376 | 16 | 16 | 4.630 | 38.067 | 0.123 | 0.480 |
| 377 | 16 | 16 | 3.133 | 38.067 | 0.003 | 0.262 |
| 378 | 16 | 16 | 5.590 | 38.067 | 0.111 | 0.488 |
| 379 | 16 | 16 | 13.750 | 38.067 | 0.818 | 0.883 |
| 380 | 16 | 16 | 2.480 | 38.067 | 0.005 | 0.296 |
| 381 | 16 | 16 | 7.900 | 38.067 | 0.189 | 0.552 |
| 382 | 16 | 16 | 2.598 | 38.067 | 0.001 | 0.201 |
| 383 | 16 | 16 | 3.775 | 38.067 | 0.059 | 0.434 |
| 384 | 16 | 16 | 3.975 | 38.067 | 0.056 | 0.411 |
| 385 | 16 | 16 | 4.200 | 38.067 | 0.112 | 0.463 |
| 386 | 16 | 16 | 2.370 | 38.067 | 0.004 | 0.267 |
| 387 | 16 | 16 | 5.990 | 38.067 | 0.490 | 0.714 |
| 388 | 16 | 16 | 2.535 | 38.067 | 0.003 | 0.226 |
| 389 | 16 | 16 | 2.760 | 38.067 | 0.019 | 0.336 |
| 390 | 16 | 16 | 9.502 | 38.067 | 0.195 | 0.539 |
| 391 | 16 | 16 | 3.412 | 38.067 | 0.011 | 0.320 |
| 392 | 16 | 16 | 4.240 | 38.067 | 0.044 | 0.411 |
| 393 | 16 | 16 | 5.432 | 38.067 | 0.016 | 0.320 |
| 394 | 16 | 16 | 4.540 | 38.067 | 0.077 | 0.426 |
| 395 | 16 | 16 | 12.967 | 38.067 | 0.143 | 0.491 |
| 396 | 16 | 16 | 19.610 | 38.067 | 0.573 | 0.754 |
| 397 | 16 | 16 | 2.933 | 38.067 | 0.011 | 0.322 |
| 398 | 16 | 16 | 11.830 | 38.067 | 0.187 | 0.525 |
| 399 | 16 | 16 | 8.630 | 38.067 | 0.267 | 0.603 |
| 400 | 16 | 16 | 3.770 | 38.067 | 0.024 | 0.354 |
| 401 | 16 | 16 | 4.790 | 38.067 | 0.052 | 0.393 |
| 402 | 16 | 16 | 2.468 | 38.067 | 0.005 | 0.272 |
| 403 | 16 | 16 | 3.945 | 38.067 | 0.017 | 0.338 |
| 404 | 16 | 16 | 3.015 | 38.067 | 0.056 | 0.411 |
| 405 | 16 | 16 | 3.933 | 38.067 | 0.038 | 0.373 |
| 406 | 16 | 16 | 4.560 | 38.067 | 0.088 | 0.473 |
| 407 | 16 | 16 | 3.267 | 38.067 | 0.090 | 0.447 |
| 408 | 16 | 16 | 7.470 | 38.067 | 0.439 | 0.699 |
| 409 | 16 | 16 | 4.845 | 38.067 | 0.162 | 0.510 |
| 410 | 16 | 16 | 2.930 | 38.067 | 0.088 | 0.466 |
| 411 | 16 | 16 | 4.118 | 38.067 | 0.022 | 0.344 |
| 412 | 16 | 16 | 2.700 | 38.067 | 0.002 | 0.264 |
| 413 | 16 | 16 | 3.128 | 38.067 | 0.002 | 0.255 |
| 414 | 16 | 16 | 4.540 | 38.067 | 0.200 | 0.536 |
| 415 | 16 | 16 | 7.990 | 38.067 | 0.343 | 0.628 |
| 416 | 16 | 16 | 4.400 | 38.067 | 0.061 | 0.424 |
| 417 | 16 | 16 | 3.575 | 38.067 | 0.017 | 0.325 |
| 418 | 16 | 16 | 5.360 | 38.067 | 0.052 | 0.415 |
| 419 | 16 | 16 | 8.670 | 38.067 | 0.201 | 0.560 |
| 420 | 16 | 16 | 4.800 | 38.067 | 0.070 | 0.431 |
| 421 | 16 | 16 | 4.133 | 38.067 | 0.088 | 0.438 |
| 422 | 16 | 16 | 7.800 | 38.067 | 0.203 | 0.544 |
| 423 | 16 | 16 | 2.517 | 38.067 | 0.002 | 0.264 |
| 424 | 16 | 16 | 2.900 | 38.067 | 0.028 | 0.387 |
| 425 | 16 | 16 | 2.433 | 38.067 | 0.003 | 0.259 |
| 426 | 16 | 16 | 4.187 | 38.067 | 0.021 | 0.356 |
| 427 | 16 | 16 | 3.160 | 38.067 | 0.030 | 0.368 |
| 428 | 16 | 16 | 4.170 | 38.067 | 0.151 | 0.498 |
| 429 | 16 | 16 | 4.050 | 38.067 | 0.035 | 0.382 |
| 430 | 16 | 16 | 5.400 | 38.067 | 0.082 | 0.450 |
| 431 | 16 | 16 | 4.550 | 38.067 | 0.123 | 0.480 |
| 432 | 16 | 16 | 3.257 | 38.067 | 0.012 | 0.325 |
| 433 | 16 | 16 | 2.730 | 38.067 | 0.161 | 0.498 |
| 434 | 16 | 16 | 3.620 | 38.067 | 0.025 | 0.365 |
| 435 | 16 | 16 | 2.535 | 38.067 | 0.002 | 0.243 |
| 436 | 16 | 16 | 2.813 | 38.067 | 0.029 | 0.371 |
| 437 | 16 | 16 | 3.553 | 38.067 | 0.027 | 0.351 |
| 438 | 16 | 16 | 2.230 | 38.067 | 0.000 | 0.186 |
| 439 | 16 | 16 | 3.102 | 38.067 | 0.026 | 0.331 |
| 440 | 16 | 16 | 5.370 | 38.067 | 0.146 | 0.505 |
| 441 | 16 | 16 | 5.285 | 38.067 | 0.037 | 0.386 |
| 442 | 16 | 16 | 5.650 | 38.067 | 0.037 | 0.379 |
| 443 | 16 | 16 | 2.967 | 38.067 | 0.030 | 0.362 |
| 444 | 16 | 16 | 2.760 | 38.067 | 0.020 | 0.345 |
| 445 | 16 | 16 | 2.710 | 38.067 | 0.077 | 0.432 |
| 446 | 16 | 16 | 10.470 | 38.067 | 0.165 | 0.534 |
| 447 | 16 | 16 | 4.990 | 38.067 | 0.100 | 0.462 |
| 448 | 16 | 16 | 2.735 | 38.067 | 0.000 | 0.186 |
| 449 | 16 | 16 | 4.510 | 38.067 | 0.057 | 0.412 |
| 450 | 16 | 16 | 2.567 | 38.067 | 0.056 | 0.405 |
| 451 | 16 | 16 | 7.000 | 38.067 | 0.257 | 0.596 |
| 452 | 16 | 16 | 4.995 | 38.067 | 0.204 | 0.563 |
| 453 | 16 | 16 | 2.880 | 38.067 | 0.002 | 0.251 |
| 454 | 16 | 16 | 2.550 | 38.067 | 0.001 | 0.225 |
| 455 | 16 | 16 | 4.050 | 38.067 | 0.004 | 0.240 |
| 456 | 16 | 16 | 3.950 | 38.067 | 0.014 | 0.326 |
| 457 | 16 | 16 | 5.770 | 38.067 | 0.140 | 0.500 |
| 458 | 16 | 16 | 3.097 | 38.067 | 0.029 | 0.373 |
| 459 | 16 | 16 | 3.228 | 38.067 | 0.016 | 0.323 |
| 460 | 16 | 16 | 4.270 | 38.067 | 0.126 | 0.481 |
| 461 | 16 | 16 | 2.400 | 38.067 | 0.002 | 0.266 |
| 462 | 16 | 16 | 5.400 | 38.067 | 0.358 | 0.631 |
| 463 | 16 | 16 | 3.015 | 38.067 | 0.031 | 0.374 |
| 464 | 16 | 16 | 6.330 | 38.067 | 0.088 | 0.452 |
| 465 | 16 | 16 | 4.185 | 38.067 | 0.063 | 0.440 |
| 466 | 16 | 16 | 3.367 | 38.067 | 0.062 | 0.422 |
| 467 | 16 | 16 | 2.140 | 38.067 | 0.001 | 0.208 |
| 468 | 16 | 16 | 2.865 | 38.067 | 0.003 | 0.272 |
| 469 | 16 | 16 | 4.670 | 38.067 | 0.027 | 0.349 |
| 470 | 16 | 16 | 2.900 | 38.067 | 0.015 | 0.318 |
| 471 | 16 | 16 | 3.750 | 38.067 | 0.079 | 0.438 |
| 472 | 16 | 16 | 2.150 | 38.067 | 0.004 | 0.276 |
| 473 | 16 | 16 | 2.967 | 38.067 | 0.074 | 0.439 |
| 474 | 16 | 16 | 5.470 | 38.067 | 0.185 | 0.529 |
| 475 | 16 | 16 | 4.235 | 38.067 | 0.139 | 0.513 |
| 476 | 16 | 16 | 6.630 | 38.067 | 0.150 | 0.497 |
| 477 | 16 | 16 | 3.100 | 38.067 | 0.057 | 0.426 |
| 478 | 16 | 16 | 2.570 | 38.067 | 0.007 | 0.308 |
| 479 | 16 | 16 | 3.197 | 38.067 | 0.005 | 0.281 |
| 480 | 16 | 16 | 3.915 | 38.067 | 0.004 | 0.262 |
| 481 | 16 | 16 | 2.250 | 38.067 | 0.001 | 0.225 |
| 482 | 16 | 16 | 2.570 | 38.067 | 0.007 | 0.295 |
| 483 | 16 | 16 | 2.683 | 38.067 | 0.002 | 0.252 |
| 484 | 16 | 16 | 7.990 | 38.067 | 0.172 | 0.518 |
| 485 | 16 | 16 | 5.180 | 38.067 | 0.098 | 0.472 |
| 486 | 16 | 16 | 5.845 | 38.067 | 0.063 | 0.448 |
| 487 | 16 | 16 | 3.725 | 38.067 | 0.002 | 0.253 |
| 488 | 16 | 16 | 4.370 | 38.067 | 0.025 | 0.360 |
| 489 | 16 | 16 | 2.900 | 38.067 | 0.003 | 0.272 |
| 490 | 16 | 16 | 4.450 | 38.067 | 0.051 | 0.407 |
| 491 | 16 | 16 | 5.400 | 38.067 | 0.062 | 0.418 |
| 492 | 16 | 16 | 2.335 | 38.067 | 0.000 | 0.190 |
| 493 | 16 | 16 | 4.700 | 38.067 | 0.002 | 0.246 |
| 494 | 16 | 16 | 7.170 | 38.067 | 0.061 | 0.416 |
| 495 | 16 | 16 | 4.685 | 38.067 | 0.026 | 0.350 |
| 496 | 16 | 16 | 2.833 | 38.067 | 0.115 | 0.464 |
| 497 | 16 | 16 | 4.290 | 38.067 | 0.023 | 0.361 |
| 498 | 16 | 16 | 4.045 | 38.067 | 0.041 | 0.403 |
| 499 | 16 | 16 | 3.215 | 38.067 | 0.001 | 0.225 |
| 500 | 16 | 16 | 3.950 | 38.067 | 0.025 | 0.343 |
| 501 | 16 | 16 | 4.133 | 38.067 | 0.171 | 0.513 |
| 502 | 16 | 16 | 2.535 | 38.067 | 0.020 | 0.361 |
| 503 | 16 | 16 | 2.850 | 38.067 | 0.003 | 0.268 |
| 504 | 16 | 16 | 2.468 | 38.067 | 0.001 | 0.247 |
| 505 | 16 | 16 | 3.695 | 38.067 | 0.144 | 0.516 |
| 506 | 16 | 16 | 3.980 | 38.067 | 0.085 | 0.448 |
| 507 | 16 | 16 | 2.883 | 38.067 | 0.011 | 0.316 |
| 508 | 16 | 16 | 6.280 | 38.067 | 0.105 | 0.482 |
| 509 | 16 | 16 | 5.135 | 38.067 | 0.026 | 0.378 |
| 510 | 16 | 16 | 3.390 | 38.067 | 0.069 | 0.448 |
| 511 | 16 | 16 | 3.197 | 38.067 | 0.039 | 0.385 |
| 512 | 16 | 16 | 2.800 | 38.067 | 0.004 | 0.276 |
| 513 | 16 | 16 | 17.653 | 38.067 | 0.619 | 0.790 |
| 514 | 16 | 16 | 4.735 | 38.067 | 0.056 | 0.434 |
| 515 | 16 | 16 | 3.390 | 38.067 | 0.262 | 0.578 |
| 516 | 16 | 16 | 4.000 | 38.067 | 0.037 | 0.358 |
| 517 | 16 | 16 | 3.095 | 38.067 | 0.020 | 0.346 |
| 518 | 16 | 16 | 2.667 | 38.067 | 0.034 | 0.386 |
| 519 | 16 | 16 | 6.100 | 38.067 | 0.427 | 0.686 |
| 520 | 16 | 16 | 3.450 | 38.067 | 0.094 | 0.452 |
| 521 | 16 | 16 | 4.270 | 38.067 | 0.170 | 0.530 |
| 522 | 16 | 16 | 7.200 | 38.067 | 0.126 | 0.492 |
| 523 | 16 | 16 | 3.100 | 38.067 | 0.036 | 0.399 |
| 524 | 16 | 16 | 10.633 | 38.067 | 0.435 | 0.676 |
| 525 | 16 | 16 | 2.882 | 38.067 | 0.001 | 0.211 |
| 526 | 16 | 16 | 5.367 | 38.067 | 0.189 | 0.553 |
| 527 | 16 | 16 | 3.000 | 38.067 | 0.026 | 0.375 |
| 528 | 16 | 16 | 3.025 | 38.067 | 0.012 | 0.326 |
| 529 | 16 | 16 | 5.200 | 38.067 | 0.321 | 0.626 |
| 530 | 16 | 16 | 3.177 | 38.067 | 0.006 | 0.263 |
| 531 | 16 | 16 | 9.100 | 38.067 | 0.389 | 0.639 |
| 532 | 16 | 16 | 3.812 | 38.067 | 0.153 | 0.505 |
| 533 | 16 | 16 | 5.900 | 38.067 | 0.192 | 0.555 |
| 534 | 16 | 16 | 3.450 | 38.067 | 0.059 | 0.415 |
| 535 | 16 | 16 | 4.752 | 38.067 | 0.148 | 0.520 |
| 536 | 16 | 16 | 6.033 | 38.067 | 0.113 | 0.476 |
| 537 | 16 | 16 | 9.200 | 38.067 | 0.097 | 0.452 |
| 538 | 16 | 16 | 2.700 | 38.067 | 0.012 | 0.321 |
| 539 | 16 | 16 | 3.700 | 38.067 | 0.073 | 0.453 |
| 540 | 16 | 16 | 2.685 | 38.067 | 0.087 | 0.464 |
| 541 | 16 | 16 | 3.805 | 38.067 | 0.028 | 0.368 |
| 542 | 16 | 16 | 3.300 | 38.067 | 0.216 | 0.558 |
| 543 | 16 | 16 | 5.430 | 38.067 | 0.035 | 0.372 |
| 544 | 16 | 16 | 4.140 | 38.067 | 0.020 | 0.313 |
| 545 | 16 | 16 | 3.167 | 38.067 | 0.019 | 0.356 |
| 546 | 16 | 16 | 4.010 | 38.067 | 0.021 | 0.353 |
| 547 | 16 | 16 | 2.285 | 38.067 | 0.050 | 0.418 |
| 548 | 16 | 16 | 5.530 | 38.067 | 0.048 | 0.393 |
| 549 | 16 | 16 | 5.282 | 38.067 | 0.059 | 0.429 |
| 550 | 16 | 16 | 7.470 | 38.067 | 0.321 | 0.618 |
| 551 | 16 | 16 | 11.433 | 38.067 | 0.521 | 0.719 |
| 552 | 16 | 16 | 4.220 | 38.067 | 0.027 | 0.371 |
| 553 | 16 | 16 | 2.305 | 38.067 | 0.001 | 0.249 |
| 554 | 16 | 16 | 2.485 | 38.067 | 0.033 | 0.374 |
| 555 | 16 | 16 | 6.630 | 38.067 | 0.111 | 0.472 |
| 556 | 16 | 16 | 3.600 | 38.067 | 0.071 | 0.413 |
| 557 | 16 | 16 | 5.530 | 38.067 | 0.209 | 0.557 |
| 558 | 16 | 16 | 2.763 | 38.067 | 0.053 | 0.408 |
| 559 | 16 | 16 | 3.220 | 38.067 | 0.009 | 0.298 |
| 560 | 16 | 16 | 2.548 | 38.067 | 0.005 | 0.305 |
| 561 | 16 | 16 | 3.650 | 38.067 | 0.013 | 0.323 |
| 562 | 16 | 16 | 3.220 | 38.067 | 0.160 | 0.502 |
| 563 | 16 | 16 | 3.867 | 38.067 | 0.054 | 0.395 |
| 564 | 16 | 16 | 4.042 | 38.067 | 0.052 | 0.422 |
| 565 | 16 | 16 | 2.570 | 38.067 | 0.004 | 0.268 |
| 566 | 16 | 16 | 3.232 | 38.067 | 0.077 | 0.460 |
| 567 | 16 | 16 | 4.400 | 38.067 | 0.096 | 0.453 |
| 568 | 16 | 16 | 3.640 | 38.067 | 0.013 | 0.329 |
| 569 | 16 | 16 | 3.217 | 38.067 | 0.025 | 0.357 |
| 570 | 16 | 16 | 3.193 | 38.067 | 0.004 | 0.270 |
| 571 | 16 | 16 | 3.100 | 38.067 | 0.046 | 0.418 |
| 572 | 16 | 16 | 3.605 | 38.067 | 0.090 | 0.449 |
| 573 | 16 | 16 | 2.467 | 38.067 | 0.042 | 0.391 |
| 574 | 16 | 16 | 5.633 | 38.067 | 0.142 | 0.514 |
| 575 | 16 | 16 | 5.100 | 38.067 | 0.060 | 0.418 |
| 576 | 16 | 16 | 4.765 | 38.067 | 0.034 | 0.386 |
| 577 | 16 | 16 | 6.300 | 38.067 | 0.044 | 0.394 |
| 578 | 16 | 16 | 2.800 | 38.067 | 0.031 | 0.354 |
| 579 | 16 | 16 | 18.030 | 38.067 | 0.260 | 0.578 |
| 580 | 16 | 16 | 5.430 | 38.067 | 0.007 | 0.265 |
| 581 | 16 | 16 | 3.017 | 38.067 | 0.004 | 0.266 |
| 582 | 16 | 16 | 4.140 | 38.067 | 0.019 | 0.327 |
| 583 | 16 | 16 | 3.305 | 38.067 | 0.032 | 0.368 |
| 584 | 16 | 16 | 8.797 | 38.067 | 0.256 | 0.574 |
| 585 | 16 | 16 | 9.350 | 38.067 | 0.242 | 0.587 |
| 586 | 16 | 16 | 3.485 | 38.067 | 0.018 | 0.363 |
| 587 | 16 | 16 | 7.000 | 38.067 | 0.366 | 0.650 |
| 588 | 16 | 16 | 7.900 | 38.067 | 0.438 | 0.691 |
| 589 | 16 | 16 | NA | 38.067 | 0.849 | 1.105 |
| 590 | 16 | 16 | 4.582 | 38.067 | 0.021 | 0.336 |
| 591 | 16 | 16 | 5.590 | 38.067 | 0.377 | 0.656 |
| 592 | 16 | 16 | 2.530 | 38.067 | 0.031 | 0.382 |
| 593 | 16 | 16 | 2.750 | 38.067 | 0.012 | 0.335 |
| 594 | 16 | 16 | 5.400 | 38.067 | 0.099 | 0.463 |
| 595 | 16 | 16 | 5.145 | 38.067 | 0.004 | 0.262 |
| 596 | 16 | 16 | 2.963 | 38.067 | 0.002 | 0.243 |
| 597 | 16 | 16 | 2.090 | 38.067 | 0.014 | 0.319 |
| 598 | 16 | 16 | 2.533 | 38.067 | 0.006 | 0.272 |
| 599 | 16 | 16 | 3.473 | 38.067 | 0.004 | 0.293 |
| 600 | 16 | 16 | 2.730 | 38.067 | 0.008 | 0.295 |
| 601 | 16 | 16 | 2.900 | 38.067 | 0.030 | 0.367 |
| 602 | 16 | 16 | 2.665 | 38.067 | 0.001 | 0.236 |
| 603 | 16 | 16 | 2.683 | 38.067 | 0.010 | 0.311 |
| 604 | 16 | 16 | 5.530 | 38.067 | 0.229 | 0.550 |
| 605 | 16 | 16 | 6.120 | 38.067 | 0.303 | 0.612 |
| 606 | 16 | 16 | 6.630 | 38.067 | 0.346 | 0.638 |
| 607 | 16 | 16 | 4.052 | 38.067 | 0.111 | 0.470 |
| 608 | 16 | 16 | 8.215 | 38.067 | 0.066 | 0.451 |
| 609 | 16 | 16 | 5.367 | 38.067 | 0.136 | 0.491 |
| 610 | 16 | 16 | 7.900 | 38.067 | 0.381 | 0.654 |
| 611 | 16 | 16 | 2.833 | 38.067 | 0.001 | 0.187 |
| 612 | 16 | 16 | 6.350 | 38.067 | 0.080 | 0.433 |
| 613 | 16 | 16 | 2.700 | 38.067 | 0.013 | 0.319 |
| 614 | 16 | 16 | 3.330 | 38.067 | 0.065 | 0.422 |
| 615 | 16 | 16 | 4.830 | 38.067 | 0.006 | 0.279 |
| 616 | 16 | 16 | 2.660 | 38.067 | 0.056 | 0.425 |
| 617 | 16 | 16 | 8.290 | 38.067 | 0.414 | 0.673 |
| 618 | 16 | 16 | 3.500 | 38.067 | 0.033 | 0.385 |
| 619 | 16 | 16 | 3.950 | 38.067 | 0.040 | 0.368 |
| 620 | 16 | 16 | 3.890 | 38.067 | 0.054 | 0.423 |
| 621 | 16 | 16 | 2.665 | 38.067 | 0.077 | 0.434 |
| 622 | 16 | 16 | 8.845 | 38.067 | 0.385 | 0.669 |
| 623 | 16 | 16 | 3.365 | 38.067 | 0.003 | 0.274 |
| 624 | 16 | 16 | 3.205 | 38.067 | 0.045 | 0.412 |
| 625 | 16 | 16 | 2.168 | 38.067 | 0.017 | 0.332 |
| 626 | 16 | 16 | 4.535 | 38.067 | 0.104 | 0.460 |
| 627 | 16 | 16 | 2.750 | 38.067 | 0.037 | 0.379 |
| 628 | 16 | 16 | 2.665 | 38.067 | 0.077 | 0.453 |
| 629 | 16 | 16 | 2.800 | 38.067 | 0.019 | 0.345 |
| 630 | 16 | 16 | 2.813 | 38.067 | 0.079 | 0.453 |
| 631 | 16 | 16 | 2.717 | 38.067 | 0.022 | 0.355 |
| 632 | 16 | 16 | 5.360 | 38.067 | 0.013 | 0.310 |
| 633 | 16 | 16 | 12.040 | 38.067 | 0.176 | 0.525 |
| 634 | 16 | 16 | 4.230 | 38.067 | 0.012 | 0.308 |
| 635 | 16 | 16 | 4.330 | 38.067 | 0.001 | 0.202 |
| 636 | 16 | 16 | 4.455 | 38.067 | 0.106 | 0.455 |
| 637 | 16 | 16 | 3.290 | 38.067 | 0.160 | 0.525 |
| 638 | 16 | 16 | 4.670 | 38.067 | 0.023 | 0.309 |
| 639 | 16 | 16 | 3.430 | 38.067 | 0.001 | 0.201 |
| 640 | 16 | 16 | 5.470 | 38.067 | 0.198 | 0.538 |
| 641 | 16 | 16 | 4.730 | 38.067 | 0.014 | 0.326 |
| 642 | 16 | 16 | 2.965 | 38.067 | 0.002 | 0.238 |
| 643 | 16 | 16 | 2.483 | 38.067 | 0.007 | 0.293 |
| 644 | 16 | 16 | 3.925 | 38.067 | 0.298 | 0.625 |
| 645 | 16 | 16 | 5.833 | 38.067 | 0.163 | 0.509 |
| 646 | 16 | 16 | 3.732 | 38.067 | 0.076 | 0.450 |
| 647 | 16 | 16 | 4.050 | 38.067 | 0.049 | 0.410 |
| 648 | 16 | 16 | 2.418 | 38.067 | 0.001 | 0.248 |
| 649 | 16 | 16 | 7.660 | 38.067 | 0.074 | 0.436 |
| 650 | 16 | 16 | 2.817 | 38.067 | 0.064 | 0.437 |
| 651 | 16 | 16 | 7.500 | 38.067 | 0.083 | 0.445 |
| 652 | 16 | 16 | 4.133 | 38.067 | 0.149 | 0.515 |
| 653 | 16 | 16 | 5.400 | 38.067 | 0.051 | 0.393 |
| 654 | 16 | 16 | 4.220 | 38.067 | 0.013 | 0.345 |
| 655 | 16 | 16 | 4.000 | 38.067 | 0.072 | 0.446 |
| 656 | 16 | 16 | 7.500 | 38.067 | 0.054 | 0.404 |
| 657 | 16 | 16 | 9.280 | 38.067 | 0.128 | 0.489 |
| 658 | 16 | 16 | 5.270 | 38.067 | 0.249 | 0.572 |
| 659 | 16 | 16 | 3.675 | 38.067 | 0.001 | 0.226 |
| 660 | 16 | 16 | 2.915 | 38.067 | 0.002 | 0.239 |
| 661 | 16 | 16 | 2.570 | 38.067 | 0.004 | 0.266 |
| 662 | 16 | 16 | 6.570 | 38.067 | 0.036 | 0.376 |
| 663 | 16 | 16 | 2.600 | 38.067 | 0.012 | 0.324 |
| 664 | 16 | 16 | 7.800 | 38.067 | 0.369 | 0.660 |
| 665 | 16 | 16 | 7.470 | 38.067 | 0.102 | 0.479 |
| 666 | 16 | 16 | 3.515 | 38.067 | 0.002 | 0.246 |
| 667 | 16 | 16 | 5.470 | 38.067 | 0.257 | 0.572 |
| 668 | 16 | 16 | 2.760 | 38.067 | 0.093 | 0.443 |
| 669 | 16 | 16 | 4.050 | 38.067 | 0.167 | 0.522 |
| 670 | 16 | 16 | 5.330 | 38.067 | 0.003 | 0.266 |
| 671 | 16 | 16 | 3.670 | 38.067 | 0.092 | 0.444 |
| 672 | 16 | 16 | 6.033 | 38.067 | 0.153 | 0.497 |
| 673 | 16 | 16 | 3.300 | 38.067 | 0.045 | 0.392 |
| 674 | 16 | 16 | 5.035 | 38.067 | 0.244 | 0.584 |
| 675 | 16 | 16 | 6.695 | 38.067 | 0.137 | 0.493 |
| 676 | 16 | 16 | 2.630 | 38.067 | 0.002 | 0.252 |
| 677 | 16 | 16 | 4.930 | 38.067 | 0.031 | 0.380 |
| 678 | 16 | 16 | 7.730 | 38.067 | 0.028 | 0.329 |
| 679 | 16 | 16 | 5.400 | 38.067 | 0.038 | 0.385 |
| 680 | 16 | 16 | 3.033 | 38.067 | 0.124 | 0.482 |
| 681 | 16 | 16 | 2.618 | 38.067 | 0.001 | 0.209 |
| 682 | 16 | 16 | 3.665 | 38.067 | 0.025 | 0.360 |
| 683 | 16 | 16 | 2.165 | 38.067 | 0.006 | 0.287 |
| 684 | 16 | 16 | 3.335 | 38.067 | 0.081 | 0.462 |
| 685 | 16 | 16 | 10.630 | 38.067 | 0.362 | 0.647 |
| 686 | 16 | 16 | 7.660 | 38.067 | 0.134 | 0.489 |
| 687 | 16 | 16 | 3.475 | 38.067 | 0.001 | 0.191 |
| 688 | 16 | 16 | 3.308 | 38.067 | 0.211 | 0.563 |
| 689 | 16 | 16 | 2.930 | 38.067 | 0.007 | 0.290 |
| 690 | 16 | 16 | 4.220 | 38.067 | 0.062 | 0.422 |
| 691 | 16 | 16 | 2.630 | 38.067 | 0.005 | 0.287 |
| 692 | 16 | 16 | 2.867 | 38.067 | 0.063 | 0.421 |
| 693 | 16 | 16 | 3.233 | 38.067 | 0.002 | 0.254 |
| 694 | 16 | 16 | 4.510 | 38.067 | 0.106 | 0.478 |
| 695 | 16 | 16 | 2.570 | 38.067 | 0.015 | 0.331 |
| 696 | 16 | 16 | 2.933 | 38.067 | 0.060 | 0.406 |
| 697 | 16 | 16 | 5.985 | 38.067 | 0.175 | 0.540 |
| 698 | 16 | 16 | 3.510 | 38.067 | 0.007 | 0.273 |
| 699 | 16 | 16 | 2.780 | 38.067 | 0.016 | 0.342 |
| 700 | 16 | 16 | 3.067 | 38.067 | 0.018 | 0.337 |
| 701 | 16 | 16 | 3.340 | 38.067 | 0.174 | 0.540 |
| 702 | 16 | 16 | 5.127 | 38.067 | 0.122 | 0.487 |
| 703 | 16 | 16 | 4.630 | 38.067 | 0.089 | 0.465 |
| 704 | 16 | 16 | 2.967 | 38.067 | 0.012 | 0.319 |
| 705 | 16 | 16 | 2.770 | 38.067 | 0.001 | 0.195 |
| 706 | 16 | 16 | 2.833 | 38.067 | 0.019 | 0.326 |
| 707 | 16 | 16 | 2.500 | 38.067 | 0.000 | 0.173 |
| 708 | 16 | 16 | 5.130 | 38.067 | 0.082 | 0.443 |
| 709 | 16 | 16 | 15.130 | 38.067 | 0.628 | 0.794 |
| 710 | 16 | 16 | 2.783 | 38.067 | 0.001 | 0.226 |
| 711 | 16 | 16 | 12.967 | 38.067 | 0.554 | 0.750 |
| 712 | 16 | 16 | 5.400 | 38.067 | 0.342 | 0.627 |
| 713 | 16 | 16 | 5.168 | 38.067 | 0.165 | 0.516 |
| 714 | 16 | 16 | 2.500 | 38.067 | 0.043 | 0.380 |
| 715 | 16 | 16 | 3.113 | 38.067 | 0.011 | 0.325 |
| 716 | 16 | 16 | 4.700 | 38.067 | 0.017 | 0.333 |
| 717 | 16 | 16 | 12.530 | 38.067 | 0.411 | 0.677 |
| 718 | 16 | 16 | 5.870 | 38.067 | 0.118 | 0.474 |
| 719 | 16 | 16 | 7.470 | 38.067 | 0.089 | 0.471 |
| 720 | 16 | 16 | 2.760 | 38.067 | 0.027 | 0.366 |
| 721 | 16 | 16 | 2.760 | 38.067 | 0.044 | 0.384 |
| 722 | 16 | 16 | 6.570 | 38.067 | 0.216 | 0.547 |
| 723 | 16 | 16 | 2.683 | 38.067 | 0.161 | 0.518 |
| 724 | 16 | 16 | 7.800 | 38.067 | 0.488 | 0.712 |
| 725 | 16 | 16 | 3.485 | 38.067 | 0.057 | 0.424 |
| 726 | 16 | 16 | 6.030 | 38.067 | 0.118 | 0.474 |
| 727 | 16 | 16 | 2.533 | 38.067 | 0.018 | 0.364 |
| 728 | 16 | 16 | 2.568 | 38.067 | 0.000 | 0.160 |
| 729 | 16 | 16 | 4.110 | 38.067 | 0.159 | 0.530 |
| 730 | 16 | 16 | 3.267 | 38.067 | 0.002 | 0.259 |
| 731 | 16 | 16 | 2.767 | 38.067 | 0.003 | 0.256 |
| 732 | 16 | 16 | 7.995 | 38.067 | 0.198 | 0.560 |
| 733 | 16 | 16 | 3.220 | 38.067 | 0.014 | 0.310 |
| 734 | 16 | 16 | 2.767 | 38.067 | 0.029 | 0.348 |
| 735 | 16 | 16 | 5.430 | 38.067 | 0.081 | 0.450 |
| 736 | 16 | 16 | 5.990 | 38.067 | 0.032 | 0.366 |
| 737 | 16 | 16 | 6.120 | 38.067 | 0.772 | 0.868 |
| 738 | 16 | 16 | 4.660 | 38.067 | 0.052 | 0.400 |
| 739 | 16 | 16 | 7.170 | 38.067 | 0.034 | 0.377 |
| 740 | 16 | 16 | 4.430 | 38.067 | 0.020 | 0.343 |
| 741 | 16 | 16 | 4.700 | 38.067 | 0.032 | 0.360 |
| 742 | 16 | 16 | 4.990 | 38.067 | 0.039 | 0.390 |
| 743 | 16 | 16 | 6.433 | 38.067 | 0.124 | 0.491 |
| 744 | 16 | 16 | 5.040 | 38.067 | 0.265 | 0.579 |
| 745 | 16 | 16 | 6.030 | 38.067 | 0.146 | 0.494 |
| 746 | 16 | 16 | 4.270 | 38.067 | 0.064 | 0.416 |
| 747 | 16 | 16 | 3.500 | 38.067 | 0.007 | 0.269 |
| 748 | 16 | 16 | 5.400 | 38.067 | 0.044 | 0.402 |
| 749 | 16 | 16 | 3.717 | 38.067 | 0.047 | 0.397 |
| 750 | 16 | 16 | 2.882 | 38.067 | 0.012 | 0.317 |
| 751 | 16 | 16 | 3.920 | 38.067 | 0.038 | 0.385 |
| 752 | 16 | 16 | 2.750 | 38.067 | 0.069 | 0.424 |
| 753 | 16 | 16 | 3.500 | 38.067 | 0.051 | 0.409 |
| 754 | 16 | 16 | 3.650 | 38.067 | 0.030 | 0.373 |
| 755 | 16 | 16 | 4.370 | 38.067 | 0.081 | 0.454 |
| 756 | 16 | 16 | 2.700 | 38.067 | 0.001 | 0.237 |
| 757 | 16 | 16 | 4.583 | 38.067 | 0.232 | 0.553 |
| 758 | 16 | 16 | 3.292 | 38.067 | 0.008 | 0.304 |
| 759 | 16 | 16 | 3.028 | 38.067 | 0.034 | 0.366 |
| 760 | 16 | 16 | 2.710 | 38.067 | 0.022 | 0.353 |
| 761 | 16 | 16 | 7.170 | 38.067 | 0.275 | 0.585 |
| 762 | 16 | 16 | 2.767 | 38.067 | 0.014 | 0.337 |
| 763 | 16 | 16 | 7.170 | 38.067 | 0.396 | 0.673 |
| 764 | 16 | 16 | 4.533 | 38.067 | 0.043 | 0.417 |
| 765 | 16 | 16 | 3.800 | 38.067 | 0.035 | 0.374 |
| 766 | 16 | 16 | 3.520 | 38.067 | 0.042 | 0.380 |
| 767 | 16 | 16 | 3.600 | 38.067 | 0.003 | 0.261 |
| 768 | 16 | 16 | 8.745 | 38.067 | 0.551 | 0.754 |
| 769 | 16 | 16 | 4.500 | 38.067 | 0.038 | 0.395 |
| 770 | 16 | 16 | 16.320 | 38.067 | 0.798 | 1.137 |
| 771 | 16 | 16 | 4.540 | 38.067 | 0.127 | 0.508 |
| 772 | 16 | 16 | 3.500 | 38.067 | 0.030 | 0.367 |
| 773 | 16 | 16 | 4.800 | 38.067 | 0.517 | 0.722 |
| 774 | 16 | 16 | 4.270 | 38.067 | 0.065 | 0.425 |
| 775 | 16 | 16 | 2.868 | 38.067 | 0.003 | 0.254 |
| 776 | 16 | 16 | 7.465 | 38.067 | 0.180 | 0.546 |
| 777 | 16 | 16 | 2.600 | 38.067 | 0.000 | 0.185 |
| 778 | 16 | 16 | 10.515 | 38.067 | 0.206 | 0.546 |
| 779 | 16 | 16 | 3.980 | 38.067 | 0.152 | 0.516 |
| 780 | 16 | 16 | 4.795 | 38.067 | 0.283 | 0.613 |
| 781 | 16 | 16 | 2.430 | 38.067 | 0.018 | 0.343 |
| 782 | 16 | 16 | 6.410 | 38.067 | 0.254 | 0.596 |
| 783 | 16 | 16 | 2.345 | 38.067 | 0.003 | 0.255 |
| 784 | 16 | 16 | 4.133 | 38.067 | 0.069 | 0.422 |
| 785 | 16 | 16 | 4.700 | 38.067 | 0.277 | 0.594 |
| 786 | 16 | 16 | 3.090 | 38.067 | 0.098 | 0.474 |
| 787 | 16 | 16 | 3.500 | 38.067 | 0.073 | 0.424 |
| 788 | 16 | 16 | 2.990 | 38.067 | 0.035 | 0.374 |
| 789 | 16 | 16 | 7.570 | 38.067 | 0.133 | 0.503 |
| 790 | 16 | 16 | 7.735 | 38.067 | 0.573 | 0.753 |
| 791 | 16 | 16 | 2.100 | 38.067 | 0.000 | 0.215 |
| 792 | 16 | 16 | 3.500 | 38.067 | 0.018 | 0.339 |
| 793 | 16 | 16 | 2.770 | 38.067 | 0.002 | 0.258 |
| 794 | 16 | 16 | 3.910 | 38.067 | 0.205 | 0.558 |
| 795 | 16 | 16 | 3.290 | 38.067 | 0.069 | 0.438 |
| 796 | 16 | 16 | 3.300 | 38.067 | 0.060 | 0.431 |
| 797 | 16 | 16 | 2.548 | 38.067 | 0.043 | 0.413 |
| 798 | 16 | 16 | 3.130 | 38.067 | 0.052 | 0.396 |
| 799 | 16 | 16 | 2.467 | 38.067 | 0.002 | 0.247 |
| 800 | 16 | 16 | 4.915 | 38.067 | 0.062 | 0.422 |
| 801 | 16 | 16 | 4.670 | 38.067 | 0.017 | 0.334 |
| 802 | 16 | 16 | 6.810 | 38.067 | 0.097 | 0.453 |
| 803 | 16 | 16 | 2.630 | 38.067 | 0.004 | 0.263 |
| 804 | 16 | 16 | 4.300 | 38.067 | 0.054 | 0.423 |
| 805 | 16 | 16 | 3.950 | 38.067 | 0.021 | 0.348 |
| 806 | 16 | 16 | 2.665 | 38.067 | 0.000 | 0.178 |
| 807 | 16 | 16 | 3.750 | 38.067 | 0.203 | 0.548 |
| 808 | 16 | 16 | 2.660 | 38.067 | 0.002 | 0.229 |
| 809 | 16 | 16 | 4.800 | 38.067 | 0.051 | 0.402 |
| 810 | 16 | 16 | 18.200 | 38.067 | 0.154 | 0.498 |
| 811 | 16 | 16 | 2.600 | 38.067 | 0.006 | 0.292 |
| 812 | 16 | 16 | 2.833 | 38.067 | 0.010 | 0.320 |
| 813 | 16 | 16 | 4.200 | 38.067 | 0.031 | 0.373 |
| 814 | 16 | 16 | 2.582 | 38.067 | 0.009 | 0.295 |
| 815 | 16 | 16 | 5.330 | 38.067 | 0.300 | 0.602 |
| 816 | 16 | 16 | 3.133 | 38.067 | 0.007 | 0.283 |
| 817 | 16 | 16 | 3.400 | 38.067 | 0.054 | 0.408 |
| 818 | 16 | 16 | 5.570 | 38.067 | 0.137 | 0.487 |
| 819 | 16 | 16 | 3.295 | 38.067 | 0.022 | 0.354 |
| 820 | 16 | 16 | 2.665 | 38.067 | 0.029 | 0.373 |
| 821 | 16 | 16 | 14.510 | 38.067 | 0.683 | 0.818 |
| 822 | 16 | 16 | 3.592 | 38.067 | 0.008 | 0.286 |
| 823 | 16 | 16 | 2.435 | 38.067 | 0.022 | 0.347 |
| 824 | 16 | 16 | 4.133 | 38.067 | 0.006 | 0.299 |
| 825 | 16 | 16 | 5.030 | 38.067 | 0.059 | 0.386 |
| 826 | 16 | 16 | 5.590 | 38.067 | 0.039 | 0.378 |
| 827 | 16 | 16 | 3.910 | 38.067 | 0.256 | 0.585 |
| 828 | 16 | 16 | 3.183 | 38.067 | 0.072 | 0.433 |
| 829 | 16 | 16 | 3.950 | 38.067 | 0.110 | 0.479 |
| 830 | 16 | 16 | 4.240 | 38.067 | 0.115 | 0.479 |
| 831 | 16 | 16 | 7.570 | 38.067 | 0.040 | 0.378 |
| 832 | 16 | 16 | 5.680 | 38.067 | 0.138 | 0.509 |
| 833 | 16 | 16 | 8.330 | 38.067 | 0.350 | 0.643 |
| 834 | 16 | 16 | 6.810 | 38.067 | 0.085 | 0.448 |
| 835 | 16 | 16 | 4.335 | 38.067 | 0.059 | 0.410 |
| 836 | 16 | 16 | 3.770 | 38.067 | 0.259 | 0.566 |
| 837 | 16 | 16 | 2.830 | 38.067 | 0.036 | 0.386 |
| 838 | 16 | 16 | 2.947 | 38.067 | 0.008 | 0.315 |
| 839 | 16 | 16 | 6.300 | 38.067 | 0.050 | 0.401 |
| 840 | 16 | 16 | 3.370 | 38.067 | 0.002 | 0.256 |
| 841 | 16 | 16 | 5.370 | 38.067 | 0.045 | 0.398 |
| 842 | 16 | 16 | 3.405 | 38.067 | 0.020 | 0.338 |
| 843 | 16 | 16 | 3.230 | 38.067 | 0.257 | 0.586 |
| 844 | 16 | 16 | 2.535 | 38.067 | 0.001 | 0.231 |
| 845 | 16 | 16 | 2.385 | 38.067 | 0.000 | 0.214 |
| 846 | 16 | 16 | 13.420 | 38.067 | 0.475 | 0.707 |
| 847 | 16 | 16 | 3.285 | 38.067 | 0.003 | 0.260 |
| 848 | 16 | 16 | 7.170 | 38.067 | 0.235 | 0.571 |
| 849 | 16 | 16 | 3.640 | 38.067 | 0.024 | 0.375 |
| 850 | 16 | 16 | 2.345 | 38.067 | 0.007 | 0.313 |
| 851 | 16 | 16 | 5.330 | 38.067 | 0.015 | 0.315 |
| 852 | 16 | 16 | 4.630 | 38.067 | 0.044 | 0.376 |
| 853 | 16 | 16 | 2.498 | 38.067 | 0.006 | 0.289 |
| 854 | 16 | 16 | 2.415 | 38.067 | 0.008 | 0.302 |
| 855 | 16 | 16 | 4.800 | 38.067 | 0.149 | 0.515 |
| 856 | 16 | 16 | 5.232 | 38.067 | 0.240 | 0.563 |
| 857 | 16 | 16 | 18.030 | 38.067 | 0.521 | 0.737 |
| 858 | 16 | 16 | 3.110 | 38.067 | 0.005 | 0.279 |
| 859 | 16 | 16 | 3.045 | 38.067 | 0.002 | 0.244 |
| 860 | 16 | 16 | 2.800 | 38.067 | 0.022 | 0.340 |
| 861 | 16 | 16 | 2.800 | 38.067 | 0.064 | 0.428 |
| 862 | 16 | 16 | 3.455 | 38.067 | 0.034 | 0.380 |
| 863 | 16 | 16 | 3.212 | 38.067 | 0.041 | 0.388 |
| 864 | 16 | 16 | 4.985 | 38.067 | 0.046 | 0.401 |
| 865 | 16 | 16 | 4.317 | 38.067 | 0.078 | 0.463 |
| 866 | 16 | 16 | 2.600 | 38.067 | 0.042 | 0.402 |
| 867 | 16 | 16 | 5.530 | 38.067 | 0.506 | 0.724 |
| 868 | 16 | 16 | 5.565 | 38.067 | 0.168 | 0.538 |
| 869 | 16 | 16 | 4.850 | 38.067 | 0.095 | 0.453 |
| 870 | 16 | 16 | 3.318 | 38.067 | 0.040 | 0.380 |
| 871 | 16 | 16 | 6.165 | 38.067 | 0.110 | 0.479 |
| 872 | 16 | 16 | 3.050 | 38.067 | 0.119 | 0.496 |
| 873 | 16 | 16 | 2.867 | 38.067 | 0.069 | 0.423 |
| 874 | 16 | 16 | 3.118 | 38.067 | 0.052 | 0.410 |
| 875 | 16 | 16 | 6.570 | 38.067 | 0.262 | 0.557 |
| 876 | 16 | 16 | 2.730 | 38.067 | 0.007 | 0.294 |
| 877 | 16 | 16 | 4.820 | 38.067 | 0.091 | 0.446 |
| 878 | 16 | 16 | 4.540 | 38.067 | 0.010 | 0.310 |
| 879 | 16 | 16 | 3.133 | 38.067 | 0.038 | 0.383 |
| 880 | 16 | 16 | 3.533 | 38.067 | 0.023 | 0.365 |
| 881 | 16 | 16 | 4.818 | 38.067 | 0.009 | 0.298 |
| 882 | 16 | 16 | 3.910 | 38.067 | 0.076 | 0.451 |
| 883 | 16 | 16 | 4.430 | 38.067 | 0.065 | 0.394 |
| 884 | 16 | 16 | 4.590 | 38.067 | 0.069 | 0.423 |
| 885 | 16 | 16 | 6.810 | 38.067 | 0.069 | 0.448 |
| 886 | 16 | 16 | 3.133 | 38.067 | 0.009 | 0.305 |
| 887 | 16 | 16 | 19.610 | 38.067 | 0.789 | 0.877 |
| 888 | 16 | 16 | 3.075 | 38.067 | 0.025 | 0.360 |
| 889 | 16 | 16 | 7.000 | 38.067 | 0.234 | 0.552 |
| 890 | 16 | 16 | 3.063 | 38.067 | 0.013 | 0.329 |
| 891 | 16 | 16 | 8.735 | 38.067 | 0.164 | 0.515 |
| 892 | 16 | 16 | 2.900 | 38.067 | 0.015 | 0.316 |
| 893 | 16 | 16 | 2.797 | 38.067 | 0.001 | 0.232 |
| 894 | 16 | 16 | 3.590 | 38.067 | 0.035 | 0.381 |
| 895 | 16 | 16 | 3.230 | 38.067 | 0.112 | 0.469 |
| 896 | 16 | 16 | 3.500 | 38.067 | 0.003 | 0.273 |
| 897 | 16 | 16 | 4.270 | 38.067 | 0.041 | 0.385 |
| 898 | 16 | 16 | 7.570 | 38.067 | 0.166 | 0.517 |
| 899 | 16 | 16 | 3.700 | 38.067 | 0.150 | 0.505 |
| 900 | 16 | 16 | 12.530 | 38.067 | 0.868 | 0.922 |
| 901 | 16 | 16 | 4.557 | 38.067 | 0.138 | 0.483 |
| 902 | 16 | 16 | 4.050 | 38.067 | 0.093 | 0.470 |
| 903 | 16 | 16 | 3.950 | 38.067 | 0.122 | 0.486 |
| 904 | 16 | 16 | 2.295 | 38.067 | 0.004 | 0.278 |
| 905 | 16 | 16 | 5.590 | 38.067 | 0.167 | 0.509 |
| 906 | 16 | 16 | 2.867 | 38.067 | 0.083 | 0.449 |
| 907 | 16 | 16 | 2.975 | 38.067 | 0.029 | 0.363 |
| 908 | 16 | 16 | 3.358 | 38.067 | 0.126 | 0.483 |
| 909 | 16 | 16 | 7.470 | 38.067 | 0.119 | 0.464 |
| 910 | 16 | 16 | 4.202 | 38.067 | 0.017 | 0.329 |
| 911 | 16 | 16 | 3.067 | 38.067 | 0.039 | 0.402 |
| 912 | 16 | 16 | 2.802 | 38.067 | 0.013 | 0.324 |
| 913 | 16 | 16 | NA | 38.067 | 0.901 | 0.937 |
| 914 | 16 | 16 | 4.885 | 38.067 | 0.265 | 0.597 |
| 915 | 16 | 16 | 3.695 | 38.067 | 0.037 | 0.401 |
| 916 | 16 | 16 | 12.530 | 38.067 | 0.592 | 0.757 |
| 917 | 16 | 16 | 2.500 | 38.067 | 0.012 | 0.324 |
| 918 | 16 | 16 | 3.500 | 38.067 | 0.311 | 0.614 |
| 919 | 16 | 16 | 4.992 | 38.067 | 0.137 | 0.512 |
| 920 | 16 | 16 | 5.530 | 38.067 | 0.016 | 0.339 |
| 921 | 16 | 16 | 3.600 | 38.067 | 0.067 | 0.440 |
| 922 | 16 | 16 | 5.100 | 38.067 | 0.217 | 0.570 |
| 923 | 16 | 16 | 4.800 | 38.067 | 0.055 | 0.406 |
| 924 | 16 | 16 | 5.330 | 38.067 | 0.057 | 0.416 |
| 925 | 16 | 16 | 3.555 | 38.067 | 0.067 | 0.425 |
| 926 | 16 | 16 | 3.930 | 38.067 | 0.014 | 0.335 |
| 927 | 16 | 16 | 3.600 | 38.067 | 0.004 | 0.269 |
| 928 | 16 | 16 | 2.660 | 38.067 | 0.001 | 0.235 |
| 929 | 16 | 16 | 2.470 | 38.067 | 0.001 | 0.223 |
| 930 | 16 | 16 | 15.600 | 38.067 | 0.580 | 0.757 |
| 931 | 16 | 16 | 1.835 | 38.067 | 0.000 | 0.131 |
| 932 | 16 | 16 | 3.800 | 38.067 | 0.167 | 0.527 |
| 933 | 16 | 16 | 4.630 | 38.067 | 0.124 | 0.490 |
| 934 | 16 | 16 | 7.500 | 38.067 | 0.725 | 0.842 |
| 935 | 16 | 16 | 3.100 | 38.067 | 0.306 | 0.609 |
| 936 | 16 | 16 | 2.100 | 38.067 | 0.007 | 0.304 |
| 937 | 16 | 16 | 2.867 | 38.067 | 0.021 | 0.326 |
| 938 | 16 | 16 | 4.510 | 38.067 | 0.578 | 0.766 |
| 939 | 16 | 16 | 4.270 | 38.067 | 0.332 | 0.611 |
| 940 | 16 | 16 | 3.390 | 38.067 | 0.020 | 0.341 |
| 941 | 16 | 16 | 3.083 | 38.067 | 0.004 | 0.279 |
| 942 | 16 | 16 | 3.670 | 38.067 | 0.038 | 0.389 |
| 943 | 16 | 16 | 2.833 | 38.067 | 0.008 | 0.318 |
| 944 | 16 | 16 | 5.450 | 38.067 | 0.095 | 0.459 |
| 945 | 16 | 16 | 5.870 | 38.067 | 0.094 | 0.436 |
| 946 | 16 | 16 | 3.160 | 38.067 | 0.336 | 0.627 |
| 947 | 16 | 16 | 4.650 | 38.067 | 0.052 | 0.403 |
| 948 | 16 | 16 | 6.170 | 38.067 | 0.149 | 0.509 |
| 949 | 16 | 16 | 5.825 | 38.067 | 0.170 | 0.537 |
| 950 | 16 | 16 | 19.110 | 38.067 | 0.387 | 0.657 |
| 951 | 16 | 16 | 3.300 | 38.067 | 0.204 | 0.546 |
| 952 | 16 | 16 | 2.600 | 38.067 | 0.002 | 0.227 |
| 953 | 16 | 16 | 2.745 | 38.067 | 0.007 | 0.299 |
| 954 | 16 | 16 | 5.370 | 38.067 | 0.087 | 0.445 |
| 955 | 16 | 16 | 3.335 | 38.067 | 0.005 | 0.277 |
| 956 | 16 | 16 | 3.227 | 38.067 | 0.016 | 0.339 |
| 957 | 16 | 16 | 3.205 | 38.067 | 0.041 | 0.411 |
| 958 | 16 | 16 | 4.270 | 38.067 | 0.007 | 0.293 |
| 959 | 16 | 16 | 2.300 | 38.067 | 0.055 | 0.415 |
| 960 | 16 | 16 | 2.780 | 38.067 | 0.039 | 0.383 |
| 961 | 16 | 16 | 3.415 | 38.067 | 0.030 | 0.372 |
| 962 | 16 | 16 | 4.690 | 38.067 | 0.153 | 0.524 |
| 963 | 16 | 16 | 4.133 | 38.067 | 0.044 | 0.412 |
| 964 | 16 | 16 | 5.590 | 38.067 | 0.300 | 0.617 |
| 965 | 16 | 16 | 3.015 | 38.067 | 0.028 | 0.382 |
| 966 | 16 | 16 | 3.950 | 38.067 | 0.014 | 0.328 |
| 967 | 16 | 16 | 3.500 | 38.067 | 0.031 | 0.349 |
| 968 | 16 | 16 | 3.720 | 38.067 | 0.031 | 0.356 |
| 969 | 16 | 16 | 2.828 | 38.067 | 0.002 | 0.250 |
| 970 | 16 | 16 | 2.650 | 38.067 | 0.009 | 0.327 |
| 971 | 16 | 16 | 4.270 | 38.067 | 0.105 | 0.480 |
| 972 | 16 | 16 | 3.100 | 38.067 | 0.008 | 0.304 |
| 973 | 16 | 16 | 3.335 | 38.067 | 0.060 | 0.437 |
| 974 | 16 | 16 | 6.733 | 38.067 | 0.179 | 0.522 |
| 975 | 16 | 16 | 5.400 | 38.067 | 0.025 | 0.377 |
| 976 | 16 | 16 | 4.080 | 38.067 | 0.007 | 0.300 |
| 977 | 16 | 16 | 3.350 | 38.067 | 0.023 | 0.326 |
| 978 | 16 | 16 | 5.655 | 38.067 | 0.036 | 0.383 |
| 979 | 16 | 16 | 11.200 | 38.067 | 0.069 | 0.427 |
| 980 | 16 | 16 | 25.000 | 38.067 | 0.548 | 0.746 |
| 981 | 16 | 16 | 4.620 | 38.067 | 0.330 | 0.629 |
| 982 | 16 | 16 | 5.400 | 38.067 | 0.075 | 0.434 |
| 983 | 16 | 16 | 4.548 | 38.067 | 0.269 | 0.592 |
| 984 | 16 | 16 | 5.430 | 38.067 | 0.239 | 0.559 |
| 985 | 16 | 16 | 2.800 | 38.067 | 0.061 | 0.439 |
| 986 | 16 | 16 | 2.520 | 38.067 | 0.086 | 0.444 |
| 987 | 16 | 16 | 2.668 | 38.067 | 0.035 | 0.399 |
| 988 | 16 | 16 | 3.950 | 38.067 | 0.012 | 0.316 |
| 989 | 16 | 16 | 10.000 | 38.067 | 0.495 | 0.723 |
| 990 | 16 | 16 | 4.105 | 38.067 | 0.023 | 0.356 |
| 991 | 16 | 16 | 3.600 | 38.067 | 0.029 | 0.373 |
| 992 | 16 | 16 | 3.093 | 38.067 | 0.006 | 0.258 |
| 993 | 16 | 16 | 2.665 | 38.067 | 0.056 | 0.406 |
| 994 | 16 | 16 | 5.407 | 38.067 | 0.025 | 0.365 |
| 995 | 16 | 16 | 4.240 | 38.067 | 0.210 | 0.528 |
| 996 | 16 | 16 | 6.675 | 38.067 | 0.259 | 0.577 |
| 997 | 16 | 16 | 6.630 | 38.067 | 0.371 | 0.656 |
| 998 | 16 | 16 | 3.165 | 38.067 | 0.026 | 0.365 |
| 999 | 16 | 16 | 3.360 | 38.067 | 0.024 | 0.340 |
| 1000 | 16 | 16 | 4.552 | 38.067 | 0.037 | 0.388 |
