# Supplementary figures and images for: Identification and validation of the association of Janus kinase 2 mutations with the response to immune checkpoint inhibitor therapy
Source: Inflamm Res. 2024 Jan 10;73(2):263–76. doi: 10.1007/s00011-023-01833-w (PMC10824873; doi:10.1007/s00011-023-01833-w)

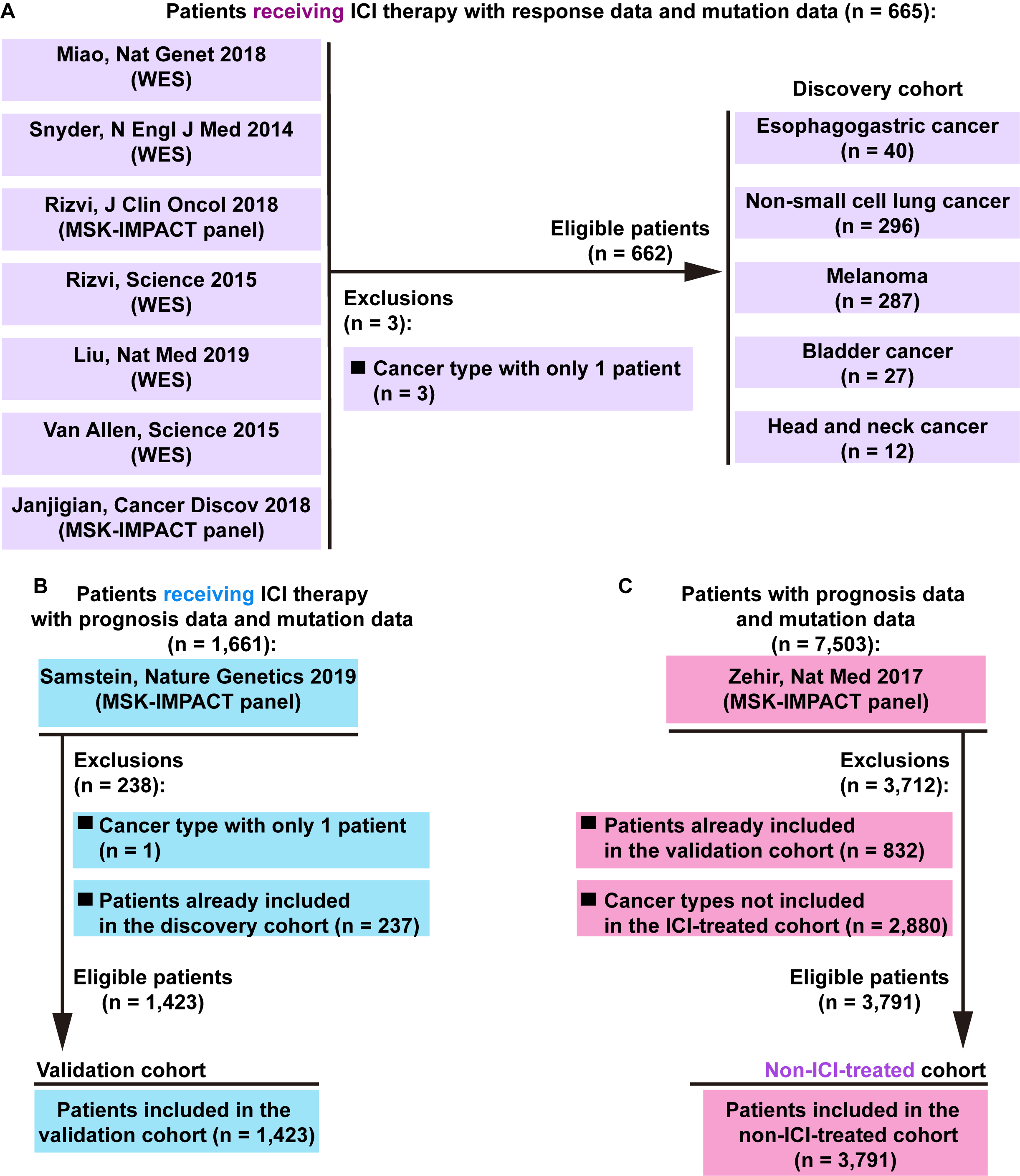

Supplement: Supplementary file 2 — Fig. S1. Process used to screen patients.(A) Overall workflow describing the process used to screen patients in the discovery cohort treated with ICI therapy. (B) Overall workflow describing the process used to screen in the verification cohort treated with ICI therapy. (C) Overall workflow describing the process for screening patients in the non-ICI cohort from the study by Zehir et al. Supplementary file 2 (TIF 1129 KB) [file 11_2023_1833_MOESM2_ESM.tif]

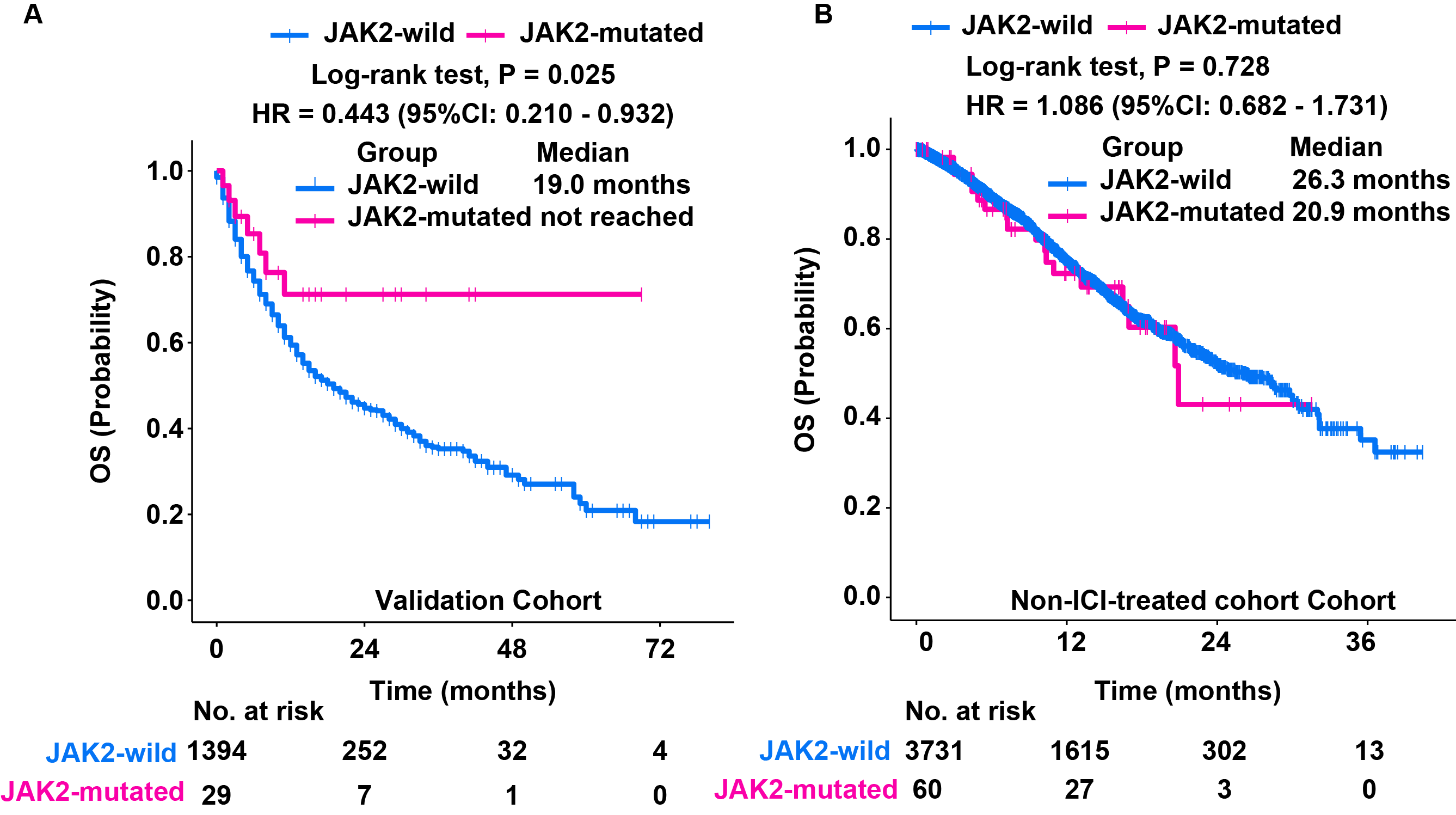

Supplement: Supplementary file 3 — Fig. S2. Overall survival of JAK2-wild and JAK2-mutated patients.(A) Kaplan-Meier curves of OS were compared between JAK2-wild and JAK2-mutated patients in the validation cohort. (B) Kaplan-Meier curves of OS were compared between JAK2-wild and JAK2-mutated patients in the non-ICI-treated cohort.Supplementary file 2 (TIF 2171 KB) [file 11_2023_1833_MOESM3_ESM.tif]
